# Photosynthetic Parameters Show Specific Responses to Essential Mineral Deficiencies

**DOI:** 10.3390/antiox10070996

**Published:** 2021-06-23

**Authors:** Miho Ohnishi, Riu Furutani, Takayuki Sohtome, Takeshi Suzuki, Shinya Wada, Soma Tanaka, Kentaro Ifuku, Daisei Ueno, Chikahiro Miyake

**Affiliations:** 1 Department of Applied Biological Science, Graduate School for Agricultural Science, Kobe University, 1-1 Rokkodai, Nada, Kobe 657-8501, Japan; miho.ohnishi02@gmail.com (M.O.); 201a521a@stu.kobe-u.ac.jp (R.F.); tksuzuki@kobe-u.ac.jp (T.S.); swada@penguin.kobe-u.ac.jp (S.W.); 203a517a@stu.kobe-u.ac.jp (S.T.); 2 Core Research for Environmental Science and Technology (CREST), Japan Science and Technology Agency (JST), 7 Gobancho, Tokyo 102-0076, Japan; saotome@bunkoukeiki.co.jp; 3 Department of System Development, Bunkoukeiki Co. Ltd., 4-8 Takakura-machi, Hachioji-shi, Tokyo 192-0033, Japan; 4 Graduate School of Agriculture, Kyoto University, Kitashirakawa Oiwake-cho, Sakyo-ku, Kyoto 606-8502, Japan; ifuku.kentaro.2m@kyoto-u.ac.jp; 5 Graduate School of Integrated Arts and Science, Kochi University, 200 Otsu, Monobe, Nankoku 783-8502, Japan; daisei_u@kochi-u.ac.jp

**Keywords:** P700, P700 oxidation system, photosynthesis, photosystem I (PSI), plant nutrition, reactive oxygen species (ROS)

## Abstract

In response to decreases in the assimilation efficiency of CO_2_, plants oxidize the reaction center chlorophyll (P700) of photosystem I (PSI) to suppress reactive oxygen species (ROS) production. In hydro-cultured sunflower leaves experiencing essential mineral deficiencies, we analyzed the following parameters that characterize PSI and PSII: (1) the reduction-oxidation states of P700 [Y(I), Y(NA), and Y(ND)]; (2) the relative electron flux in PSII [Y(II)]; (3) the reduction state of the primary electron acceptor in PSII, Q_A_ (1 − qL); and (4) the non-photochemical quenching of chlorophyll fluorescence (NPQ). Deficiency treatments for the minerals N, P, Mn, Mg, S, and Zn decreased Y(II) with an increase in the oxidized P700 [Y(ND)], while deficiencies for the minerals K, Fe, Ca, B, and Mo decreased Y(II) without an increase in Y(ND). During the induction of photosynthesis, the above parameters showed specific responses to each mineral. That is, we could diagnose the mineral deficiency and identify which mineral affected the photosynthesis parameters.

## 1. Introduction

Yield increases for main crops, such as cassava, corn, potatoes, rice, soybeans, and wheat, are required to help support our increasing global population. Bioengineering methods to increase growth potentials have included the introduction of critical phenotypes, such as those for environmental stress tolerances, including drought, higher and lower temperatures, and salinity, both alone and in combination. This has been done using traditional breeding and cutting-edge molecular biology techniques that use specific genes to convey tolerance. In the present study, we have proposed a new method to diagnose nutrient deficiencies in the early stages of plant growth, prior to the typical symptoms of mineral deficiencies that occur in intact plant leaves. This could enable us to improve the growth conditions of crops before they suffer damage that may lead to retarded growth. This method is based on the physiological responses of photosynthetic parameters, as described below. All oxygenic photosynthetic organisms show the same response of suppressing reactive oxygen species in chloroplasts.

C3-plants, angiosperms, show a tight coupling of their light and dark reactions, including both the Calvin–Benson–Bassham cycle (CBB cycle) and the photorespiratory carbon-oxidation pathway (photorespiration) in their net CO_2_ assimilation [1,2,3,4,5,6,7]. The chemical energy compounds nicotinamide adenine dinucleotide phosphate (NADPH), ferredoxin (Fd), and adenosine triphosphate (ATP), which are produced in the light reaction of the photosynthetic electron transport system, drive the CBB cycle to regenerate ribulose 1,5-bisphosphate (RuBP), one of the substrates of RuBP carboxylase/oxygenase (Rubisco), and photorespiration, which also contributes to the regeneration of RuBP. Under atmospheric conditions, Rubisco catalyzes both the carboxylation and oxygenation of RuBP, which are the primary reactions of both the CBB cycle and photorespiration. The dark reactions use Fd and NADPH as reductants for the regeneration of RuBP and drive the Fd photo-reduced oxidation reaction in photosystem I (PSI) [7,8]. Furthermore, the dark reactions use ATP produced in the catalytic reaction of ATP synthase [6,9,10,11], which consumes the proton motive force induced in the light reaction [12,13]. These are the molecular mechanisms that support the tight coupling of the light reaction with dark reactions in net CO_2_ assimilation [5,6]. While the light reaction drives the dark reaction, it is inversely, caused by a dark reaction.

The tight coupling of the light and dark reactions carries a potential risk of oxidative damage. Net CO_2_ assimilation is suppressed by exposure to environmental stresses, such as drought, extreme low/high temperatures, and nutrient deficiencies [14,15]. The suppression of dark reactions lowers the utilization of the Fd, NADPH, and ATP produced in the light reaction, which could directly affect light reactions by reducing the use efficiency of the electrons produced in the photosynthetic electron transport system [14,16,17]. As a result, the accumulation of electrons in the photosynthetic electron transport system can quickly cause PSI photoinactivation [18]. In the dark, the repetitive short-pulse (rSP) illumination treatment for the leaves of angiosperm plants selectively inactivates PSI but not PSII [18,19]. The accumulation of electrons in PSI induced by the rSP-illumination treatment enhances superoxide radical production, which is a reactive oxygen species (ROS) [14,20]. The inactivation of PSI in C3-plants, such as Arabidopsis, barley, cucumber, potato, and spinach, has been reported when stress conditions cause electrons to accumulate in the photosynthetic electron transport system [21,22,23,24,25,26,27,28,29,30,31,32,33,34,35,36,37,38,39,40].

Oxygenic photosynthetic organisms have acquired PSI protection mechanisms from ROS attacks during their evolution [14,18,41]. The scavenging system of ROS (the water-water cycle) functions near PSI in the thylakoid membranes. Superoxide dismutase (SOD), ascorbate peroxidase (APX), monodehydroascorbate radical reductase (MDAR), dehydroascorbate reductase (DHAR), and glutathione reductase (GR) contribute to the removal of superoxide radicals and hydrogen peroxide [14,16,17,42,43,44,45]. Furthermore, all oxygenic photosynthetic organisms have mechanisms to suppress ROS production in PSI [18,46]. Under illuminated conditions, the rSP-illumination treatment does not inactivate PSI in the intact leaves of angiosperms [18]. Actinic light illumination oxidizes the reaction center chlorophyll P700 in PSI [18]. P700 is then photoexcited to P700* and donates electrons to the electron carriers in PSI, producing oxidized P700 (P700^+^). P700^+^ is then reduced to the ground state by electrons from photosystem II (PSII). The turnover of P700 in the photo-oxidation reduction cycle catalyzes the electron flow from plastocyanin to Fd in PSI. The accumulation of P700^+^ in the cycle reduces the ratio of P700*, which can donate electrons to O_2_, producing superoxide radicals [5,6]. This is the suppression mechanism of ROS in PSI, which all oxygenic photosynthetic organisms have [18,46].

As described above, in C3-plants, the electron flux in the light reaction (or the dark reaction) has a negative linear relationship with the existence probability of P700^+^ [11,18,46]. This is a physiological response of PSI, which lowers the efficiency of photosynthesis to suppress ROS production. We have focused on the phenomenon of P700 oxidation for the usage of the new biomarker for the stress degree in plants, which can be measured non-destructively. There has been a large amount of research on P700 oxidation in PSI, and specifically, for photosynthetic organisms exposed to conditions that reduce photosynthetic efficiency, such as high light, low temperature, and drought [47,48,49,50,51,52,53,54,55]. With regard to the nutrient deficiencies, however, there is insufficient information on how these stresses influence the photosynthetic electron transport reaction. In the present study, we tested P700 oxidation in sunflower leaves exposed to various nutrient deficiencies. Some nutrient deficiencies decrease photosynthetic activity, and prolonged deficiencies can lower productivity and reduce plant yields [15,56,57,58]. Sunflower plants were cultivated from seedlings under control conditions and then transferred to nutrient-deficient conditions. We then confirmed the robustness of P700 oxidation in response to the suppressed photosynthesis activity due to nutrient deficiencies at the steady state, except for the B, Ca, Fe, K, and Mo deficiencies. We found that the photosynthetic parameters of PSII and PSI had diverse responses during the induction period of photosynthesis in response to the nutrient deficiencies. Based on these facts, we propose that photosynthetic parameters can be utilized for the early diagnosis of nutrient deficiencies in the future.

## 2. Materials and Methods

### 2.1. Plant Materials and Growth Conditions

Sunflower (*Helianthus annuus*) plants were grown in a controlled chamber (14 h light at 27 °C/10 h dark at 25 °C; light intensity 300–400 µmol photons m^−2^ s^−1^; relative humidity 50–60%). Seeds of sunflower, purchased from TAKII & Co., Ltd. (Kyoto, Japan), were germinated in flowing tap water for five days in the controlled chamber. Five-day-old seedlings were planted in pots filled with a hydroponic solution of 1/2 strength of Hoagland solution for three days. The plants were then transferred to the hydroponic solution of the original Hoagland solution for four days. The composition of the original Hoagland solution contained the following macronutrients [NH_4_NO_3_ (2 mM), KH_2_PO_4_ (0.8 mM), CaCl_2_ (0.6 mM), and MgSO_4_ (0.5 mM)]; and micronutrients [H_3_BO_4_ (50 µM), MnSO_4_ (9 µM), ZnSO_4_ (0.7 µM), CuSO_4_ (0.3 µM), Na_2_MoO_4_ (0.25 µM), and NaFeEDTA (45 µM)]. The pH of the nutrient solution was adjusted to 5.3–5.5, by adding 10 mM MES (2-morpholinoethanesulfonic acid) during cultivation. The plants were then transferred to the original Hoagland solution deprived of essential minerals (N, P, K, S, Ca, Zn, Mo, B, Fe, Mn, Cu, and Mg) for one week. Then, in the one-week period after the one week of exposure to the treatment, the measurements of each sample were taken. The solutions were renewed once a week and always aerated with air. For all experiments, we measured the second leaves from the top of the plants.

### 2.2. Measurements of PSI and PSII Parameters

P700^+^ absorbance and chlorophyll fluorescence were simultaneously measured using a Dual-PAM/F instrument (Walz, Effeltrich, Germany) with a closed leaf-type chamber (Bunkoukeiki Co., Ltd., Tokyo, Japan), in which expiratory air (assumed to be CO_2_ saturated air) was filled and the temperature was set at 25 ± 0.1 °C using a Peltier controller system (Bunkoukeiki Co., Ltd. Tokyo, Japan). Leaf discs (3.4 cm^2^) from dark-adapted sunflowers were placed in the chamber and actinic light (AL; 630 nm, 650 µmol m^−2^ s^−1^) was applied to the leaves.

The transmittance changes in P700^+^ were estimated. The photosynthetic parameters of PSI were obtained as follows [59]: the relative amount of maximal photo-oxidizable P700 by saturated pulse illumination (SP, 300 ms of duration, 16,500 µmol photons m^−2^ s^−1^) under AL, Y(I) = Pm’/Pm, the relative amount of oxidized P700 under AL, Y(ND) = P/Pm; the rest state of P700 under AL, which reflects the electron pressure at the acceptor side of P700, Y(NA) = (Pm–Pm’)/Pm: Pm, total amount of photo-oxidizable P700; Pm’, the maximal photo-oxidizable P700 by SP illumination under AL; and P, the photo-oxidized P700 under AL. For the determination of Pm, SP illumination was applied after 10 s of illumination with far-red light (820 nm). 

The photosynthetic parameters of PSII were calculated from the chlorophyll fluorescence as follows [60]: the maximal quantum yield of PSII, Fv/Fm = (Fm − Fo)/Fm the effective quantum yield of PSII, Y(II) = (Fm’ − Fs)/Fm; the ratio of photo-induced non-photochemical quenching of absorbed light energy, Y(NPQ) = [(Fm − Fm’)/Fm’]’ (Fs/Fm); the fraction of closed chlorophyll center of PSII, which reflects the reduction level of Q_A_, the primary electron acceptor of PSII, (1 – qL) = 1 – (Fm’ − Fs)/(Fm’ − Fo’)’ (Fo’/Fs) or = 1 – [Y(II)/(1 − Y(II))]’ [(1 − (Fv/Fm))/(Fv/Fm)]’ (Fm/Fm’) [61]: Fm, the maximal chlorophyll fluorescence of dark-adapted leaves under SP illumination; Fo, the basal level of chlorophyll fluorescence only under weak pulse-modulated measuring light illumination (ML, 620 nm, 0.2 µmol photons m^−2^ s^−1^); Fm’, the maximal chlorophyll fluorescence under AL; Fs, the stationary level of chlorophyll fluorescence under AL; and Fo’, the basal level of chlorophyll fluorescence under AL. 

In this study, we calculated the original parameters for diagnosis as follows; Y(ND)/Y(II)_N_, Y(NA)/Y(II)_N_, Y(NPQ)/Y(II)_N_, and (1 − qL)/Y(II)_N_. Subscript N means “normalized”, where the values of these parameters were divided by the maximum values.

### 2.3. Analysis of Minerals in Plants

A raw leaf blade (3.4 cm^2^) was homogenized in 50 mM sodium-phosphate buffer (pH 7.2) containing 120 mM 2-mercaptoethanol, 1 mM iodoacetic acid, and 5% (*v/v*) glycerol at a leaf:buffer ratio of 1:9 (g/mL) in a chilled mortar and pestle. The total chlorophyll (Chl) and leaf nitrogen contents were measured from a part of this homogenate. Absorbances at 720, 663, and 645 nm were measured to calculate the chlorophyll content by the Arnon method. The Chl content in leaves was represented on a leaf-area basis [62]. The total leaf nitrogen content was determined using Nessler’s regent in a digestion solution after the addition of potassium sodium tartrate [63]. The homogenate was decomposed by 60% (*v*/*v*) sulfuric acid and 30% (*v*/*v*) H_2_O_2_ with heat. The decomposing leaf solution was mixed with distilled water, 10% (*w*/*v*) potassium sodium tartrate solution, and 2.5 N NaOH, and Nessler’s reagent was immediately added to the mixture. The nitrogen content was determined by measuring the change in the absorbance at 420 nm.

Oven-dried and finely cut leaves were subjected to HNO_3_/HClO_4_ digestion on a block digester (Digi PREP MS, SCP SCIENCE, Baie-d’Urfé, Quebec, QC, Canada) for elemental analysis of the leaves. The concentrations of K, Ca, Zn, Mo, Fe, Mn, and Mg in each digestion solution were analyzed using inductively coupled plasma mass spectrometry (ICP-MS NEXION300D, PerkinElmer, Waltham, MA, USA), and P, B, Cu, and S in each digestion solution were analyzed using inductively coupled plasma optical emission spectrometry (ICP-OES SPS3100, Hitachi High-Tech Science, Tokyo, Japan).

### 2.4. Analysis of the Minerals in the Soils

The soil pH was determined using a glass electrode in a 1:2.5 ratio of the soil to solution mixture. Soil electrical conductivity (EC) was determined using an EC electrode in a 1:5 ratio of soil to water mixture. Available nitrogen was determined using the phosphate buffer extraction method [64]. Available phosphorous was determined using the Trough method [65]. Exchangeable K, Mg, and Ca were determined using the ammonium acetate extraction method [66]. Available iron, zinc, manganese, and copper were determined using the DTPA-TEA extraction method [67]. Easily reducible manganese was determined using the ammonium acetate-hydroquinone solution extraction method [68].

### 2.5. Statistical Analysis

Statistical analyses of the corresponding data in Supplementary Figure S1 and Supplementary Tables S1–S4 (ANOVA, ANCOVA, Student’s *t*-test, and Dunnett’s test) were performed using the commercial software JMP8 (ver. 14.2.0, SAS Institute Inc. Cary, NC, USA). The number of biological replicates was 3–4.

## 3. Results

### 3.1. Characterization of the Nutrient Deficiency-Treated Plants

We examined the effects of nutrient deficiencies for each of the following essential minerals: N, P, K, S, Ca, Zn, Mo, B, Fe, Mn, Cu, and Mg, in the intact leaves of sunflower plants (Supplementary Table S1). Each mineral deficiency treatment decreased the corresponding mineral content in the leaves when compared with the control plants. We then analyzed the nutrient deficiency-treated plants. 

The essential mineral deficiencies had different effects on the chlorophyll content, based on the leaf area of the sunflower plants (Supplementary Table S2). The Ca, B, Fe, Mg, Mn, N, and S deficiencies significantly decreased the chlorophyll content when compared to the control plants (*p* < 0.05).

The essential mineral deficiencies also affected the nitrogen content in the sunflower leaves (Supplementary Table S2). The Ca- and K-deficiency treatments increased the nitrogen content, while the N deficiency-treated plants significantly decreased the nitrogen levels compared with the control plants (*p* < 0.05).

The essential mineral deficiencies affected the maximum quantum yield of PSII (Fv/Fm) in the sunflower plants (Supplementary Table S2). Plants treated with Mg and Mn deficiencies had significantly decreased Fv/Fm values when compared to the control plants (*p* < 0.05). The other nutrient-deficient plants showed no effects on the Fv/Fm values.

The essential mineral deficiencies affected the maximum photo-oxidizable P700 content (Pm) in the sunflower leaves (Supplementary Table S2). Plants treated with Fe, Mg, Mn, N, and S deficiencies had significantly decreased Pm in comparison with the control (*p* < 0.05). 

### 3.2. Steady-State Analysis of P700 Oxidation

**Figure 1:**
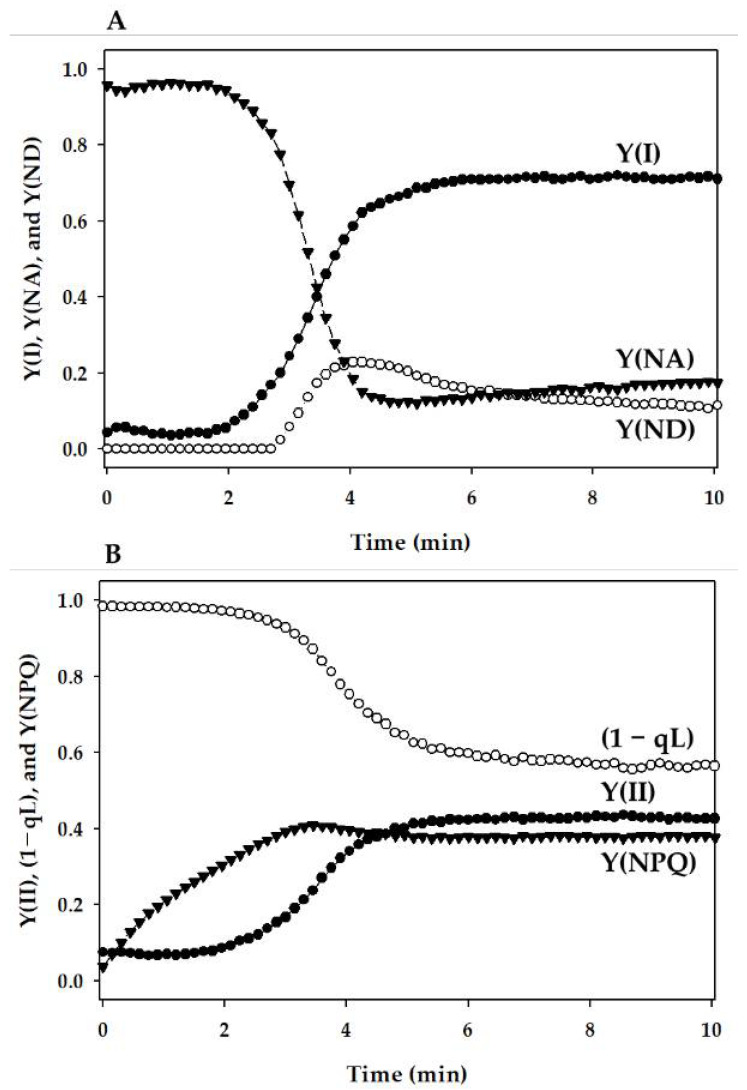
Induction of photosynthesis parameters for photosystems I (PSI) and II (PSII) of the intact leaves of the sunflower plants (*Helianthus annuus*). (**A**) PSI parameters: Y(I), Y(NA), and Y(ND) were plotted against the actinic light (AL) illumination time. (**B**) PSII parameters: Y(II), Y(NPQ), and (1 − qL) were plotted against the AL illumination time. The leaves were illuminated with AL of 1000 µmol photons m^−2^ s^−1^ in the presence of 1% CO_2_ and 20% O_2_ in the chamber (25 °C). AL was turned on at 0 min. Each data set presented is a typical representation.

To understand the response of the P700 redox state to the nutrient deficiencies, we measured the changes in chlorophyll fluorescence parameters: Y(II), Y(NPQ), (1 − qL), and the P700 redox states: Y(I), Y(NA), and Y(ND) during the induction of photosynthesis (Figure 1).

**Figure 2:**
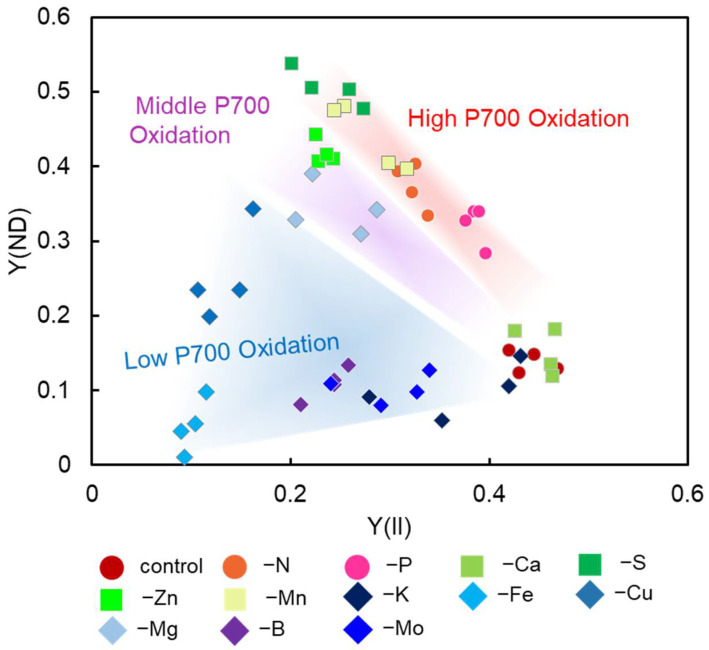
Relationships between Y(ND) and Y(II). The data were from the induction analysis of photosynthesis parameters, as shown in Figure 1, and the steady-state values of both Y(II) and Y(ND) were plotted. Sunflower plants (*Helianthus annuus*) were grown under the nutrient-deficiency conditions, as follows. Circles: red, control; orange, nitrogen deficiency (–N); pink, phosphate deficiency (–P). Diamonds: dark blue, potassium deficiency (–K); blue, iron deficiency (–Fe); purple, copper deficiency (–Cu); pale blue, magnesium deficiency (–Mg); dark purple, boron deficiency (–B); medium purple, molybdenum deficiency (–Mo); pale green, calcium deficiency (–Ca); dark green, sulfur deficiency (–S); green, zinc deficiency (–Zn); pale-green, manganese deficiency (–Mn). Sunflower plants (*Helianthus annuus*) were grown in the fields, as follows. Responses of Y(ND) against Y(II) were divided into three types: High P700 Oxidation, Middle P700 Oxidation, and Low P700 Oxidation. Please see the details in the text for more information (in Section 3.2).

We plotted the steady-state values of Y(ND) against Y(II) for the leaves of the sunflower plants, which were cultivated under nutrient-deficient conditions (Figure 2). Viewed as a whole, we divided the distribution of the plots into three groups: high P700 oxidation, middle P700 oxidation, and low P700 oxidation. The high P700 oxidation group consisted of the N, P, Mn, Ca, and S deficiency-treated sunflower plants (Figure 2, Supplementary Figure S1). The middle P700 oxidation group consisted of the Zn and Mg deficiency-treated sunflower plants (Figure 2, Supplementary Figure S1). The high and middle oxidations for the P700 were statistically discriminated into two groups using ANCOVA analysis (*p* < 0.05) (Supplementary Figure S1). The low P700 oxidation group consisted of Fe, K, Mo, B, and Cu and showed the lowest increase in P700 oxidation, even with a decrease in Y(II) (Figure 2). In other words, the P700 oxidation system did not function. 

### 3.3. Induction Analysis of the Photosynthesis Parameters for PSI and the Electron Flux in PSII

**Figure 3:**
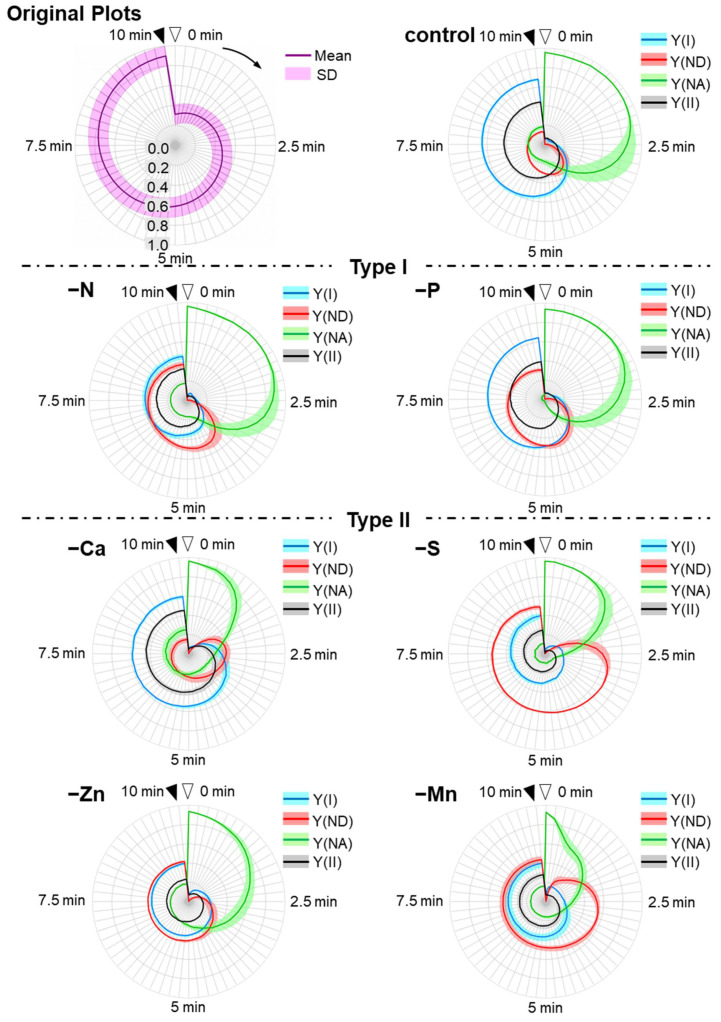

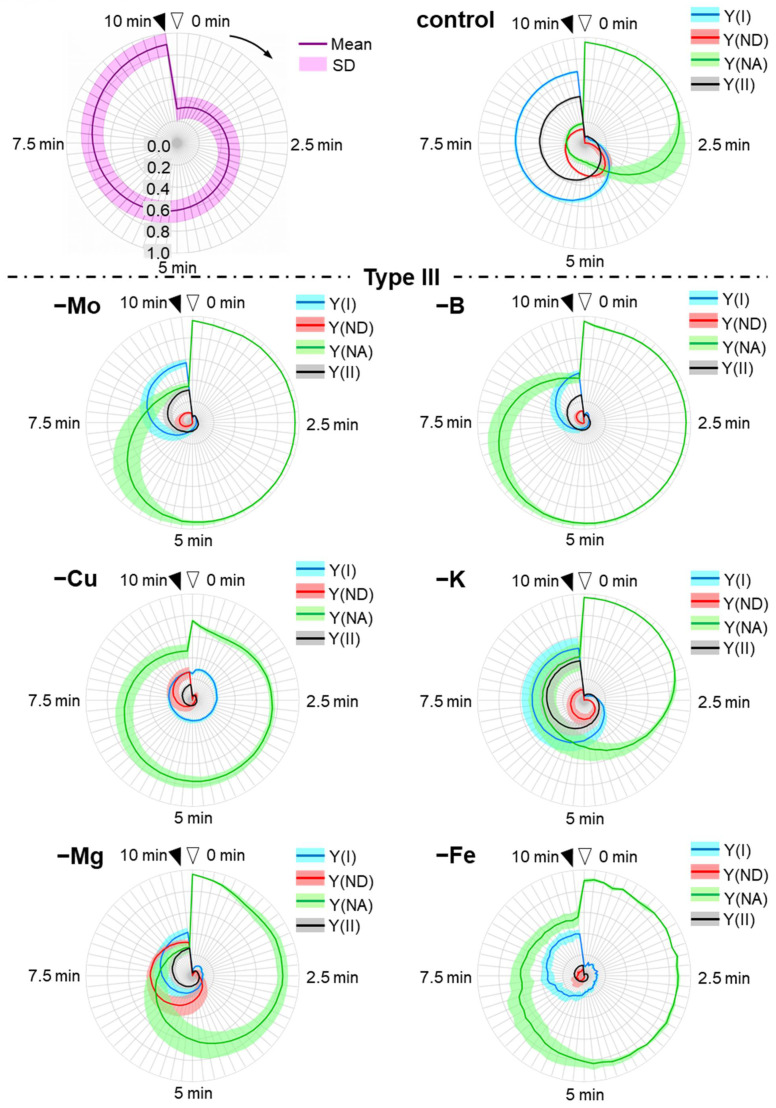
Effects of the nutrient-deficiency treatments on the induction of the photosynthesis parameters for PSI and PSII in the intact leaves of sunflower plants (*Helianthus annuus*). Data are taken from Figure 1. These parameters [Y(I), blue; Y(ND), red; Y(NA), green; Y(II), black] during the induction of photosynthesis were plotted against the AL illumination time in the radar-chart plots (Original plots). Nutrient-deficiency treatments are follows: Type-I: nitrogen deficiency (–N); phosphate deficiency (–P). Type-II: calcium deficiency (–Ca); zinc deficiency (–Zn); sulfur deficiency (–S); manganese deficiency. Type-III: molybdenum deficiency (–Mo); copper deficiency (–Cu); magnesium deficiency (–Mg); boron deficiency (–B); potassium deficiency (–K); iron deficiency (–Fe). Data are shown as mean values (line) + standard deviation (shadow) and were calculated from the data collected from our four experiments.

The P700 redox state parameters showed different responses depending on the nutrient-deficiency treatments, during the induction of photosynthesis after actinic light exposure (AL, 650 µmol photons m^−2^ s^−1^) (Figure 3). Y(I) shows the existing probability of the ground state of P700 in its photo-oxidation reduction cycle during saturated-pulse illumination. Y(ND) shows the existing probability of the oxidized form of P700 and the extent of the reduction of P700^+^ in the photo-oxidation reduction cycle of P700. Y(NA) shows the existing probability of the photoexcited state of P700 and the extent of the oxidation reaction of P700* in the photo-oxidation reduction cycle of P700 [59]. Y(II) shows the relative electron flux in PSII and the photosynthetic linear electron flow activity [60].

**Figure 4:**
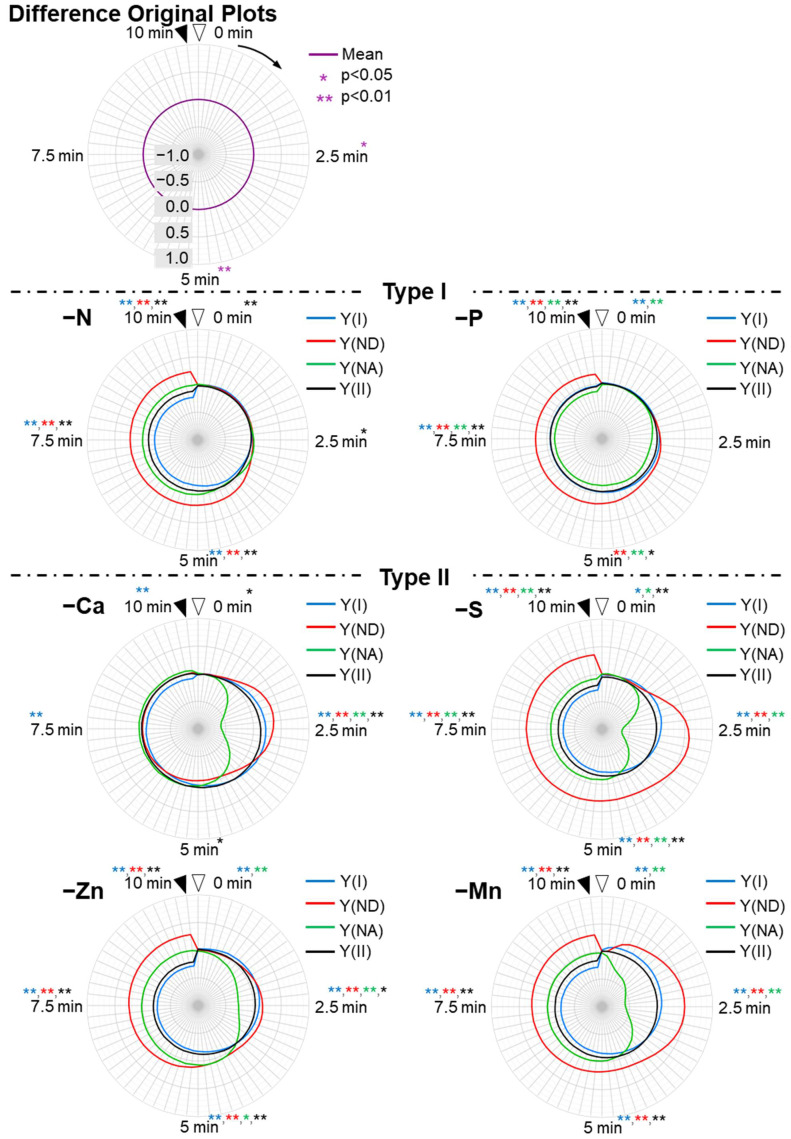

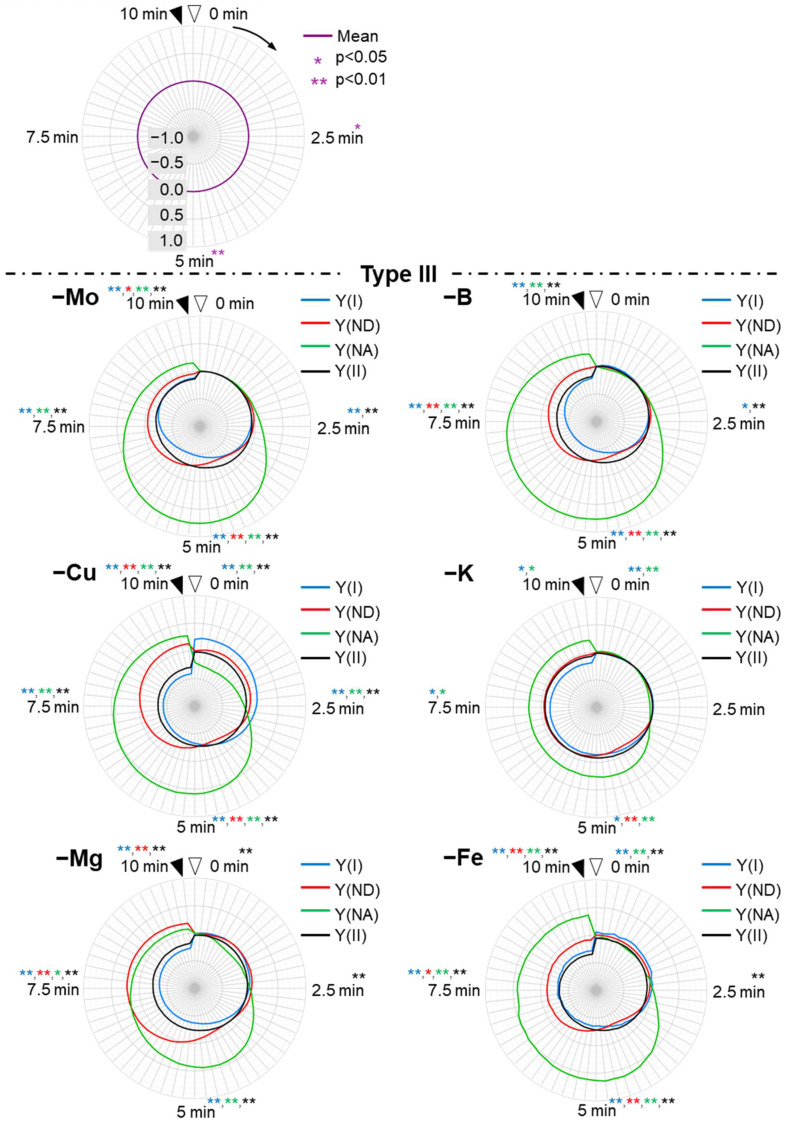
Effects of nutrient-deficiency treatments on the photosynthesis parameters of PSI and PSII in the intact leaves of sunflower plants (*Helianthus annuus*). Data were calculated from Figure 3. The parameters [Y(I), blue; Y(ND), red; Y(NA), green; Y(II), black] were the difference values between the control and the nutrient deficiency (difference original plots). Nutrient-deficiency treatments are as follows: Type-I: nitrogen deficiency (–N); phosphate deficiency (–P). Type-II: calcium deficiency (–Ca); zinc deficiency (–Zn); sulfur deficiency (–S); manganese deficiency. Type-III: molybdenum deficiency (–Mo); cupper deficiency (–Cu); magnesium deficiency (–Mg); boron deficiency (–B); potassium deficiency (–K); iron deficiency (–Fe). Data presented are the differences of the means between the control plot and each mineral-deficiency plot, and were calculated from the data collected from our four experiments. The results of statistical treatments between the means of the four parameters at 0, 2.5, 5, 7.5, and 10 min by Student’s *t*-test are presented in each panel and Supplementary Table S3. * and ** denote statistically significant differences at *p* < 0.05 and *p* < 0.01, respectively. The colors of the asterisks correspond to those of the four parameters described above.

To compare the responses of these parameters to the nutrient deficiencies, we plotted them against time on the radar chart after the AL was turned on (original plots, Figure 3). Plus, we plotted the differences of the original parameters between control plants and nutrient-deficient plants in order to extract the characteristics of the influences on those parameters by the nutrients-deficiency treatments (difference original plots, Figure 4).

In the control plants, Y(NA) rapidly reached the maximum value, and this was sustained for approximately 2.5 min. Then, the photosynthetic linear electron flow started to increase, as observed in Y(II). With the increase in Y(ND), Y(I) became more extensive than Y(II). At the steady state, Y(I) was much higher than Y(II), and Y(II) was more extensive than Y(ND).

N and P deficiency-treated plants showed the same Y(NA)-relief time as the control plants, but each parameter value was different from that of the control plants (Control induction time type: Type-I). In type I plants, Y(NA) values at 2.5 min were not significantly different from in control plants (*p* > 0.05), and 5 min or later after the illumination, Y(NA) values were not significantly different or smaller when compared to the control plants (Figure 3 and Figure 4 and Supplementary Table S3). In the N deficiency-treated plants, both Y(II) and Y(I) significantly decreased after 5 min of illumination when compared to those in the control plants (Supplementary Table S3). Instead, Y(ND) increased and complemented the decrease in Y(I), which had the same tendency in its Y(NA) behavior as the control plants (*p* > 0.05, Supplementary Table S3). In the P deficiency-treated plants, Y(ND) was largely induced after 5 min of illumination, which was larger than that in the control plants (*p* < 0.01, Supplementary Table S3). Y(I) and Y(II) slightly decreased. Therefore, the increase in Y(ND) suppressed Y(NA) to nearly zero, which is a distinctive feature of the P deficiency-treated plants. At the steady state, Y(II) was almost the same as Y(ND). Y(NA) decreased and disappeared after approximately 7 min.

Unlike both the control and the P and N deficiency-treated plants, the nutrient (Ca, Zn, Mn, and S)-deficient plants showed suppressed Y(NA) in the first quarter (0–2.5 min) (Rapid Y(NA) relief type: Type-II). This feature can be seen as the hollow shapes of Y(NA) at around 2.5 min in difference original plots (Figure 4), where the Y(NA) values of type II plants were significantly smaller than that in control plants (*p* < 0.01, Supplementary Table S3). This could be due to the rapid induction of both Y(ND) and Y(I) (*p* < 0.01, Supplementary Table S3). In the Ca deficiency-treated plants, at the steady state, Y(I) was more extensive than Y(II), and Y(II) was more extensive than Y(ND), which is similar to that in the control plants. However, a rapid induction of Y(II), Y(I), and Y(ND) was observed, which suppressed Y(NA) in the first quarter. In both the Zn and Mn deficiency-treated plants, Y(I) was the same as Y(ND), both of which were larger than Y(II). In the Mn-deficient plants, the most rapid induction of Y(ND) and suppression of Y(NA) was observed. In the Zn-deficient plants, the induction of Y(I), Y(II), and Y(ND) were the slowest among the rapid Y(NA) relief-type plants. Although the S deficiency-treated plants showed suppressed Y(NA), which could be due to the enhanced Y(ND), Y(ND) was more extensive than Y(I), and Y(I) was more extensive than Y(II) at the steady state.

The rest of the nutrient (Mo, B, Cu, Fe, Mg, and K) deficiency-treated plants showed higher Y(NA) values than control plants especially after 5 min (*p* < 0.05, Supplementary Table S3) (High Y(NA) type: Type III). In both the Mo- and B-deficient plants, Y(NA) was highly enhanced, and the higher values were maintained after 5 or 7.5 min of AL illumination. This could be due to the delayed induction of both Y(II), Y(I), and Y(ND). In the Mo-deficient plants, Y(I) was more extensive than Y(II), and Y(II) was more extensive than Y(ND) at the steady state. In contrast to the Mo deficiency-treated plants, for the B deficiency-treated plants, Y(NA) was more extensive than the values for Y(I), Y(II), and Y(ND) for 10 min during the induction of photosynthesis. Like the B deficiency-treated plants, for the Cu deficiency-treated plants, the Y(NA) was larger than the other parameters; however, Y(NA) was lower than that in the B deficiency-treated plants. Moreover, the initial value of the Y(NA) was the lowest (approximately 0.7), and was only observed in Cu-deficient treatments. This was due to the rapid induction of Y(I) (Figure 3, Supplementary Table S3). Y(I) increased as soon as the AL was turned on. In the Fe deficiency-treated plants, the behaviors of the parameters resembled those in the Cu deficiency-treated plants. Both Mg and K deficiency-treated plants showed mild Y(NA), which started to decrease after 4–5 min. Mg deficiency-treated plants showed the highest values for Y(ND) among the high Y(NA)-type plants. K deficiency-treated plants showed the highest values of both Y(I) and Y(II). 

### 3.4. Induction Analysis of the Parameters Focusing on the Redox Levels of Plastoquinone and P700 in PSI, and Non-Photochemical Quenching of Chlorophyll Fluorescence against the Electron Flux in PSII

**Figure 5:**
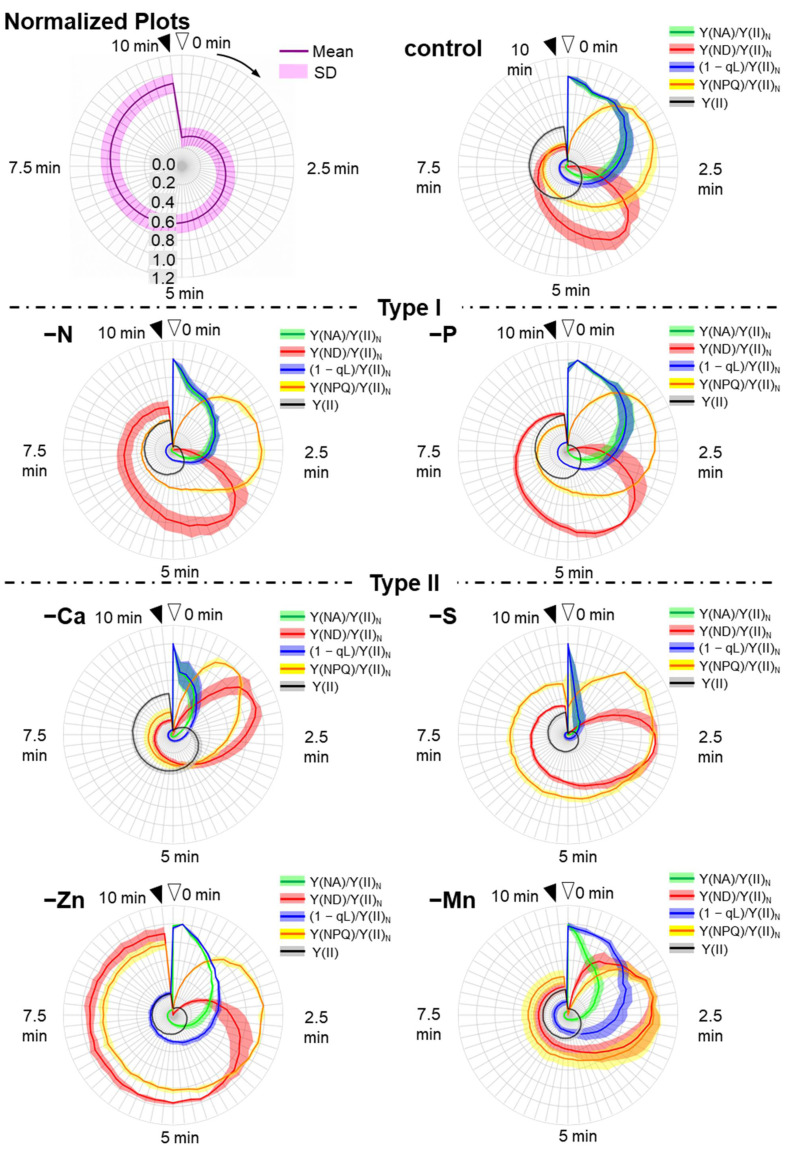

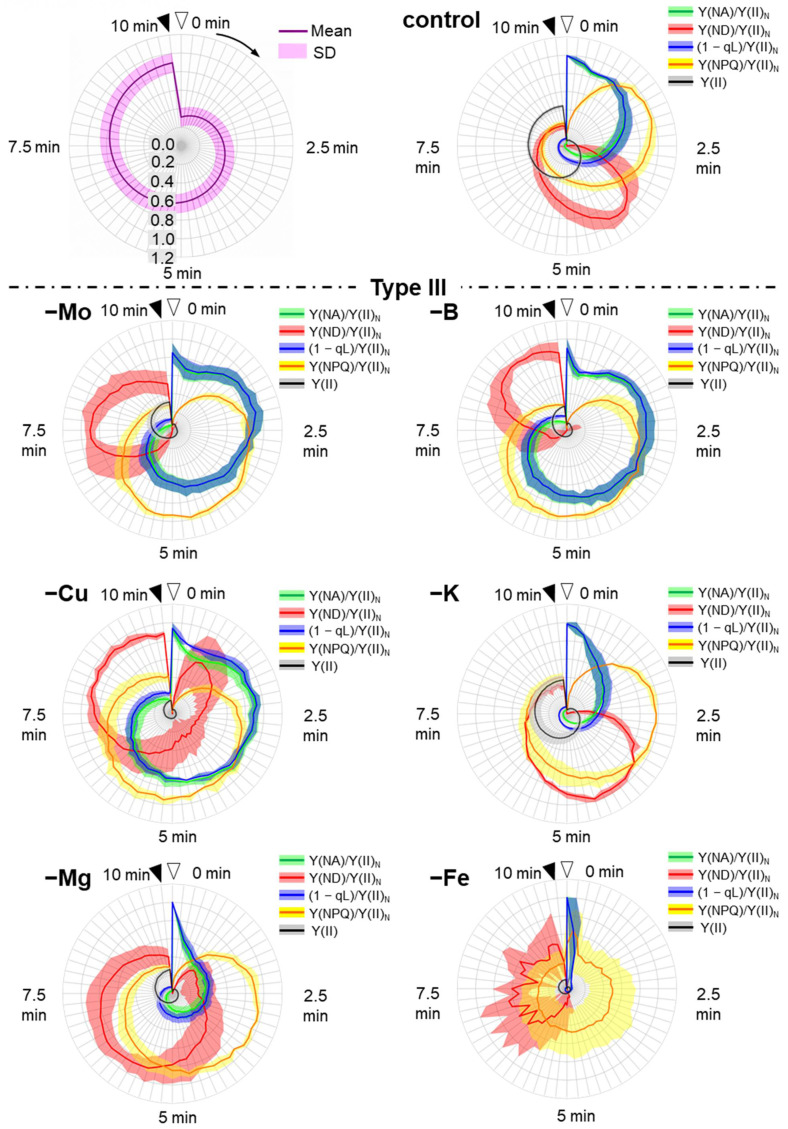
Effects of nutrient-deficiency treatments on the photosynthesis parameters of both photosystems PSI and PSII in the intact leaves of sunflower plants (*Helianthus annuus*). Data were calculated from Figure 3. The parameters [Y(NA)/Y(II)_N_, green; Y(ND)/Y(II)_N_, red; (1 − qL)/Y(II)_N_, blue; Y(NPQ)/Y(II)_N_, yellow; Y(II), black] during the induction of photosynthesis were plotted against the AL illumination time in the radar-chart plots (Normalized plots). These parameters were normalized by being divided by their maximal values, except for Y(II). Nutrient-deficiency treatments were as follows: Type-I: nitrogen deficiency (–N); phosphate deficiency (–P). Type-II: calcium deficiency (–Ca); zinc deficiency (–Zn); sulfur deficiency (–S); manganese deficiency. Type-III: molybdenum deficiency (–Mo); cupper deficiency (–Cu); magnesium deficiency (–Mg); boron deficiency (–B); potassium deficiency (–K); iron deficiency (–Fe). Data are shown as mean values (line) + standard deviation (shadow) and were calculated from the data collected from our four experiments.

**Figure 6:**
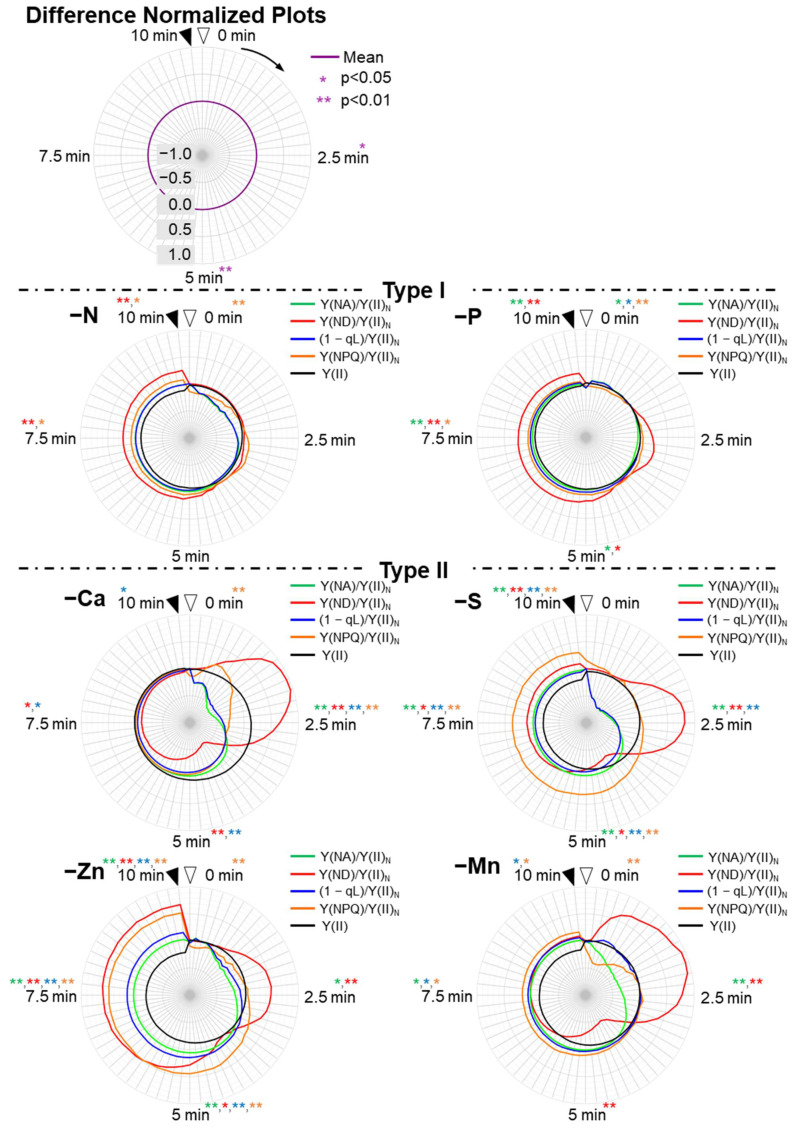

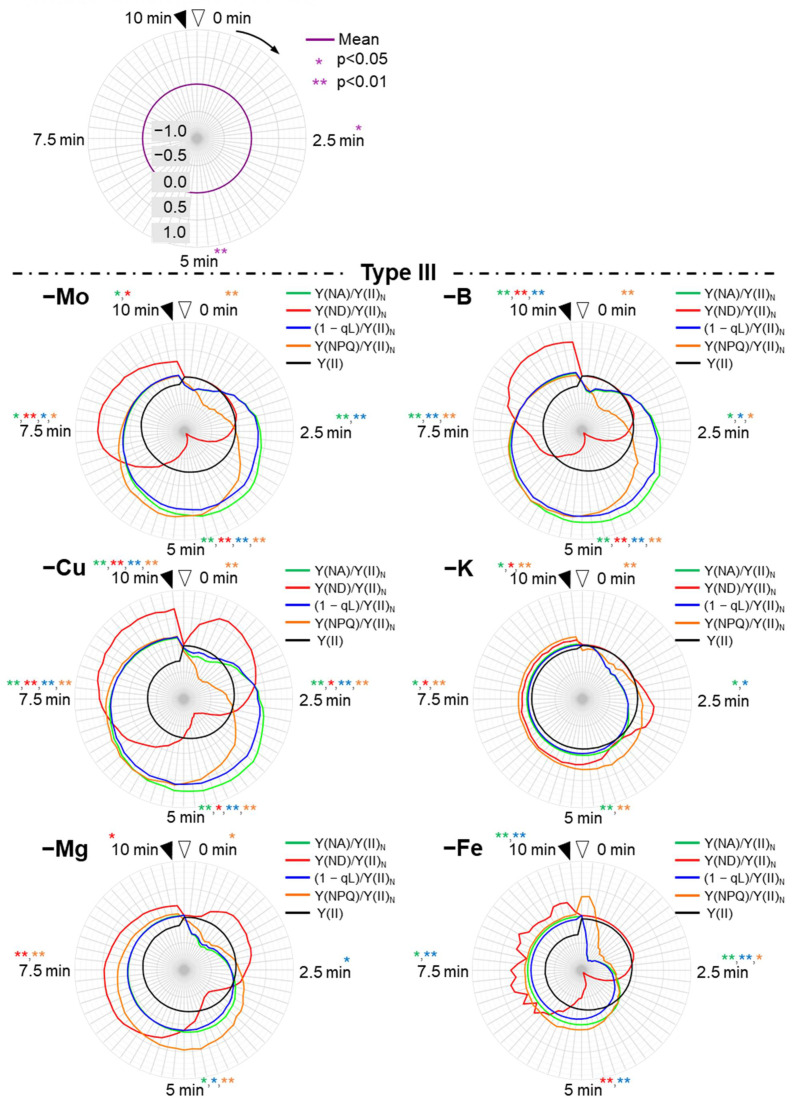
Effects of nutrient-deficiency treatments on the photosynthesis parameters of PSI and PSII in the intact leaves of sunflower plants (*Helianthus annuus*). Data were calculated from Figure 4. The parameters [Y(NA)/Y(II)_N_, green; Y(ND)/Y(II)_N_, red; (1 − qL)/Y(II)_N_, blue; Y(NPQ)/Y(II)_N_, yellow; Y(II), black] were the differences between the control and the nutrient deficiency (difference normalized plots). Nutrient-deficiency treatments were as follows: Type-I: nitrogen deficiency (–N); phosphate deficiency (–P). Type-II: calcium deficiency (–Ca); zinc deficiency (–Zn); sulfur deficiency (–S); manganese deficiency. Type-III: molybdenum deficiency (–Mo); cupper deficiency (–Cu); magnesium deficiency (–Mg); boron deficiency (–B); potassium deficiency (–K); iron deficiency (–Fe). Data were the differences in the mean between the control plot and each mineral-deficiency plot, which were calculated from the data collected from our four experiments. The results of statistical treatments between the means of the four parameters at 0, 2.5, 5, 7.5, and 10 min by Student’s *t*-test are presented in each panel and Supplementary Table S3. * and ** denote statistically significant differences at *p* < 0.05 and *p* < 0.01, respectively. The colors of the asterisks correspond to those of the four parameters described above.

As described above, the chlorophyll fluorescence and P700 redox state parameters showed different responses to the different nutrient-deficiency treatments during the induction of photosynthesis. To extract the unique responses of these parameters among the nutrient-deficient plants, we used four parameters: [Y(ND)/Y(II)_N_], [Y(NA)/Y(II)_N_], [Y(NPQ)/Y(II)_N_], and [(1 − qL)/Y(II)_N_]. All parameters were normalized by dividing with the maximal values of each parameter in each nutrient-deficiency treatment. Y(NPQ) shows the extent of the non-photochemical quenching of chlorophyll fluorescence in PSII [60]. The (1 − qL) shows the extent of the reduction state for the plastoquinone pool, where the redox level of the primary quinone acceptor (Q_A_) in PSII is assumed to be in rapid equilibrium with the redox level of the plastoquinone pool [60,61]. We plotted these normalized parameters and the differences of them between control and nutrient-deficient plants in the induction phases of photosynthesis (Figure 5 and Figure 6).

We found different responses of the normalized parameters in the same type when compared to those in the original plots (Figure 3 and Figure 5). Even within the type II plants, we found significantly different behaviors of the normalized parameters. Ca-deficient plants showed the most rapid inductions of those parameters, where all four normalized parameters showed the peaks at 0–2.5 min (Figure 3). S-deficient plants showed the narrowest shapes of Y(NA)/Y(II)_N_ and (1 − qL)/Y(II)_N_ because the Y(II) values at 0 min were almost zero, which make Y(NA)/Y(II) and (1 − qL)/Y(II) values at 0 min exceedingly high. Zn deficiency-treated plants showed expanded Y(ND)/Y(II)_N_ and Y(NPQ)/Y(II)_N_, which means peak-less behaviors of these parameters. Mn-deficient plants showed the most significant separation between Y(NA)/Y(II)_N_ and (1 − qL)/Y(II)_N_ at 0–2.5 min when compared to the other mineral-deficiency treatments. 

In type III, except for Cu deficiency-treated plants, the induction of either Y(ND)/Y(II)_N_ or Y(NPQ)/Y(II)_N_ was largely delayed, unlike in both Type I and II (Figure 5). Cu-deficient plants showed a rapid peak of Y(ND)/Y(II)_N_ at 1 min, which was earlier than Y(NPQ)/Y(II)_N_, and expanded Y(NA)/Y(II)_N_ and (1 − qL)/Y(II)_N_ values. Mg-deficient plants also showed the small peak of Y(ND)/Y(II)_N_ around 1 min. However, it showed rapid relief of Y(NA)/Y(II)_N_ and (1 − qL)/Y(II)_N_ unlike Cu-deficient plants. Mo and B deficiency-treated plants showed expanded Y(NA)/Y(II)_N_ and (1 − qL)/Y(II)_N_ like Cu-deficient plants but showed the first peaks of Y(ND)/Y(II)_N_ after 7.5 min, not around 0–2.5 min. These two nutrient-deficiency treatments led to similar behaviors of the parameters. Still, we found the difference in Y(ND)/Y(II)_N_ around 10 min, which reduced to 0.5 in Mo deficient-treated plants while it remained high in B-deficient plants. K-deficient plants showed control-like behavior of the normalized parameters. However, we found a statistical difference in those parameters, which were higher than those of control plants after 5 min of illumination (Figure 6, Supplementary Table S4). Fe deficiency-treated plants showed noisy behavior of Y(ND)/Y(II)_N_ because both Y(II) and Y(ND) were severely suppressed throughout the illumination. Moreover, Fe-deficient plants showed the narrowest shapes of Y(NA)/Y(II)_N_ and (1 − qL)/Y(II)_N_ for the same reason as S-deficient plants.

## 4. Discussion

In the present study, we found that the deficiencies in essential minerals decreased the apparent quantum yield of PSII [Y(II)], with an increase in the oxidation level of P700 [Y(ND)]. There was an exception, however, for the following minerals: K, Fe, Ca, B, and Mo. The negative relationship between Y(ND) and Y(II) was considered robust. However, the deficiencies of K, Fe, Ca, B, and Mo destroyed this (Figure 2). These catastrophes of this robustness were due to the enhanced Y(NA), Type-III, for K, Fe, B, and Mo deficiencies. Furthermore, we found that the diversity of the photosynthetic parameter inductions, as shown in the original plots, depended on the essential mineral deficiencies. Furthermore, we found that higher values of Y(ND)/Y(II) could be a physiological marker of poor growth unless the cultivation conditions were improved. We call the physiological marker Y(ND), the ROS marker, because P700 oxidation suppresses ROS production [18].

The changes in the photosynthetic parameters Y(NPQ), (1 − qL), Y(II), and Y(ND) were assessed using the P700 oxidation mechanism in PSI (Supplementary Figure S2) [5]. Furthermore, the behaviors of both Y(NA) and Y(I) can be described using the turnover of the photo-oxidation reduction cycle of P700 during saturated-pulse (SP) illumination [5,6].

The photosynthetic parameters in the present study were determined as follows (Supplementary Figure S2). Y(II) reflects the electron flux of the photosynthetic linear electron flow (Jf) or the electron consumption rates in both net CO_2_ assimilation and photorespiration (Jg), and Jf is equal to Jg [1,2,3,4]. Y(NPQ) reflects NPQ, which is determined by proton motive force (pmf). As shown in Supplementary Figure S2, pmf is regulated by gH^+^, Jf [12,13], and JgH^+^ [9]. JgH^+^ indicates the consumption rate of pmf. The pmf consumption rate is equal to JgH^+^ = gH^+^ × pmf = kH^+^ × Jf [5,6]; then, pmf = (kH^+^ × Jf)/gH^+^ = JgH^+^/gH^+^. Generally, the decrease in the net CO_2_ assimilation is reflected as a decrease in Y(II), accompanied by an enhanced decrease in gH^+^, compared to the decreases in both (kH^+^ × Jf) and JgH^+^ [7,11]. The pmf subsequently increases. Furthermore, Jf = kqL × (1 − qL) = Jg [11,46]. Then, (1 − qL) = Jf/kqL = Jg/kqL. Generally, the decrease in the net CO_2_ assimilation is reflected as a decrease in Y(II), accompanied by an enhanced decrease in kqL, compared to the decreases in both Jf and Jg [5,6]. kqL reflects the apparent rate constant of the oxidation activity of PQH2 by the Cyt b_6_/f-complex. Therefore, the suppression of net CO_2_ assimilation increased in both pmf and (1 − qL). Increased pmf levels induce NPQ to decrease Y(II) and lower the oxidation activity of reduced plastoquinone by the Cyt b_6_/f-complex [69,70,71]. Simultaneously, the enhanced reduction of plastoquinone, observed as the increase in (1 − qL), lowers the Q-cycle activity of the Cyt b_6_/f-complex (RISE) [11,72,73,74,75]. The suppression of the electron fluxes in both PSII and the Cyt b_6_/f-complex causes the reduction reaction of P700^+^ to be the rate-determining step of the P700 photo-oxidation reduction cycle in PSI, which increases Y(ND) [5,6]. The proportions of Y(I), Y(NA), and Y(ND) depend on the rate-determining step of the P700 photo-oxidation reduction cycle during SP illumination. Practically, the values of both Y(I) and Y(NA) were determined using the SP illumination of the leaves, where the maximum photo-oxidizable P700 (Pm’) was estimated. Importantly, the values for Pm’ depend on the oxidation level of P700, that is, the P700^+^ values [5,6]. At the steady state, the induction of P700^+^ shows the limitation of the reduction reaction of P700^+^ in the P700 photo-oxidation reduction cycle. Then, the Pm’ value can be overestimated by SP illumination [6]. As described above, by using the P700 oxidation model [5], we could understand the behavior of the photosynthetic parameters, as shown in both the original and normalized plots.

We observed the typical responses of a photosynthetic electron transport reaction depending on the 12 kinds of nutrient-deficiency treatments. 

N-deficient plants, which were classified into type-I, showed suppressed Y(I) and Y(II), which indicated the lowered electron-sink activity probably because of the lack of an N source for biosynthesis of Rubisco, which is the largest destination of fixed nitrogen (Figure 3 and Figure 4) [63]. The imbalanced electron sink-source size leads to the enhancement of pmf, which contributes to the oxidation of P700, as we showed above. 

P-deficiency plants also belonged to type-I (Figure 3). P is involved in ATP synthesis as one of the substrates and phosphorylation of the light-harvesting chlorophyll protein complexes II (LHCII) in the photosynthetic electron transport chain resulting in the state transition [76,77]. We estimate that P deficiency would suppress ATP synthesis, which caused strong acidification of the luminal side of the thylakoid membrane and suppressed Y(NA) to almost zero (Figure 3).

Mn is literally one of the components of the Mn_4_CaO_5_ cluster at PSII, which is the site of photo-oxidation of water [78]. Previously, it was reported that a 33-kD protein fragment in oxygen-evolving complex (OEC33) possesses carbonic anhydrase (CA) activity and the inhibition of CA activity of OEC33 lowers the maximal quantum yield of PSII in the isolated thylakoid membrane [79]. Moreover, it was suggested that Mn would be the co-factor of this CA activity, unlike other CAs [80]. Actually, in Mn-deficient plants, the maximal quantum yields of PSII (Fv/Fm) was severely suppressed (Supplementary Table S2). Therefore, Mn deficiency would lead to a lowered supply of electrons from PSII to PSI, while it would result in relatively small influences on the Calvin–Benson cycle (CBC), which caused the rapid suppression of Y(NA) (Figure 3). 

Ca also contributes to the function of OEC, stabilizing the redox potential and the interaction around the Mn_4_CaO_5_ cluster [81,82]. Therefore, Ca-deficient plants also showed type II behavior for the same reason as the Mn deficiency. However, the contribution of Ca to the function of OEC is relatively minor when compared to Mn [83], which gave higher Y(II) and Y(I) values in Ca-deficient plants than those in Mn deficiency-treated plants (Figure 3). 

S composes sulfur-containing amino acids and iron sulfur clusters. The disulfide linkages in some thiol enzymes of CBC, such as glyceraldehyde-3-phosphate dehydrogenase (GAPDH), fructose 1,6-busphosphatase (FBPase), sedoheptulose-1,7-bisphosphatase (SBPase) and phosphoribulokinase (PRK), the γ-subunit of ATP synthetase, and the redox regulator protein, thioredoxin, are the redox state-dependent switch of their activity [10,84]. S-deficient plants showed a different behavior from Fe-deficient plants, although Fe is also the essential component of iron-sulfur proteins (Figure 3). Therefore, it could be estimated that there would be a malfunction of thioredoxin-mediated redox regulation in S-deficient plants. However, it remains unclear why the rapid relief of Y(NA) and high Y(ND) were observed in S-deficient plants (Figure 3). 

Zn is one of the components of a lot of enzymes in plants, including the carbonic anhydrase and Cu-Zn superoxide dismutase (SOD) [85]. Considering the CO_2_ saturated condition in our measurements, Zn deficiency did not seem to influence the CO_2_ supply to Rubisco. We could not estimate how numerous Zn deficiency-induced metabolic dysfunctions caused the specific response of photosynthetic parameters (Figure 3).

Mg is one of the components of chlorophyll and is essential for the activation of Rubisco [86,87]. Therefore, Mg-deficient plants showed lowered Fv/Fm (Supplementary Table S2) and prolonged induction of the photosynthetic parameters (Figure 3). 

K, one of the three major nutrients for plants, is involved in the leaf anatomy [87,88], ionic equilibrium at intramembrane, and the activation of carboxylation activity of Rubisco [88,89]. It was reported that the anatomical alteration in leaves by K deficiency lowered the mesophyll conductance [90]. In the current study, photosynthetic measurements were carried out under CO_2_ saturated conditions. Therefore, the influences of a K deficiency on photosynthetic parameters are caused by other reasons, such as the delay of the activation of Rubisco and the malfunction of the ΔpH-induced regulation of the electron transport reaction, which could cause the high-Y(NA) symptom (Figure 3).

Cu is essential for the function of PC, the electron donor of PSI [91]. Actually, the Cu-deficient plants showed a low amount of photo-active PC (10% of the control plants), measured by Dual KLAS-NIR (Heinz Walz, Effeltrich, Germany) (data not shown; see [92] for the methods). We estimated that the low Y(NA) values at 0 min (approximately 0.7, Figure 3), which was the specific response observed only in Cu-deficient plants, were due to the lack of an electron donor of PSI. However, it is still unclear why such a retarded relief of Y(NA) was observed (Figure 3).

Fe is essential in the photosynthetic apparatus as the component of the electron carriers, such as F_x_, F_A_/F_B_, and Fd at the acceptor side of PSI, and Cyt *b*_L_/*b*_H_, rieske Fe-S cluster and Cyt *f* at Cyt *b_6_f* complex [93,94]. Moreover, proto IX monomethyl ester cyclase and chlorophyllide *a* oxygenase, the enzymes in the chlorophyll biosynthesis pathway, contain an iron-sulfur cluster [95]. Therefore, the chlorophyll content in leaves was significantly decreased in Fe-deficient plants (Supplementary Table S2). In Fe-deficient plants, Y(ND) was suppressed to nearly zero and Y(NA) remained high throughout the illumination (Figure 3). Therefore, it could be estimated that the iron-sulfur electron acceptors of PSI would be suppressed in Fe-deficient plants. This strong acceptor-side limitation of PSI led to significant photoinhibition of PSI, as we can see in the decrease in the Pm value of Fe-deficient plants (about 20% of control plants) (Supplementary Table S2).

B is not directly involved in the photosynthetic apparatus. B is known for the components of the cell walls and contributes to the stabilization of the cell walls by borate cross-linking of pectin [96]. Therefore, B deficiency could influence the mesophyll conductance. Considering the CO_2_ saturated conditions in our measurements, however, the typical response of B-deficient plants cannot be fully explained (Figure 3).

Mo is also not directly involved in the photosynthetic apparatus, and is one of the components for nitrate reductase. It is known that Mo deficiency suppresses the activity of nitrate reductase, resulting in the accumulation of nitrate in plants [97]. It could be estimated that the accumulation of nitrate would somehow suppress the photosynthetic reaction, which would cause such a retarded induction of photosynthetic parameters (Figure 3).

Combining original plots and normalized plots (supplementary with the difference plots, Fv/Fm and Pm) (Figure 3, Figure 4, Figure 5 and Figure 6 and Supplementary Table S2), we confirmed that each mineral-deficient plant showed different features in the photosynthetic parameters during the induction period of photosynthesis. Importantly, we confirmed the high reproducibility of the diverse responses of the photosynthetic parameters to nutrient deficiencies in sunflower plants. If we observed the typical response to the essential mineral deficiency, we could identify which minerals were deficient during the cultivation of plants and crops. Under field conditions, plants and crops would be exposed to the deficiencies and sometimes an excess of several types of essential minerals at the same time [98]. More studies should be advanced on the applicability of this method to field-grown plants, and moreover, on the detailed mechanisms of how each mineral deficiency influences the photosynthetic electron transport reaction, directly and indirectly.

## 5. Conclusions

In the present research, we confirmed the typical responses of photosynthetic parameters in the 10-min induction period of photosynthesis depending on 12 kinds of nutrient deficiencies. The feasibility of the diagnosis method of the nutrient condition in plants and crops was strongly suggested by the non-destructive measurements of photosynthetic parameters, which took only 10 min. For the future study, the applicability of this method needs to be tested in field-grown plants exposed to the more complicated nutrient condition [98], and more research should be conducted on the detailed mechanisms of how each nutrient deficiency influences the photosynthetic electron transport reaction.

## Data Availability

Data is contained within the article.

## Supplementary Materials

The following are available online at https://www.mdpi.com/article/10.3390/antiox10070996/s1. Supplemental Figure S1. Grouping of nutrient-deficiency treatments in Y(II)-Y(ND) relationships. Supplemental Figure S2. Regulation of electron and H^+^ fluxes in photosynthesis in response to the activities of both CO_2_ assimilation and photorespiration in C3 plants. Supplemental Table S1. Essential mineral content in the mineral deficiency-treated sunflower leaves. Supplemental Table S2. Chlorophyll content, nitrogen content, Fv/Fm, and Pm in the sunflower leaves treated with mineral deficiencies. Supplemental Table S3. Statistical analysis for the differences of the parameters in original plots between control and the mineral deficiency-treated plants at 0, 2.5, 5, 7.5 and 10 min after illumination. Supplemental Table S4. Statistical analysis for the differences of the parameters in normalized plots between control and the mineral deficiency-treated plants at 0, 2.5, 5, 7.5 and 10 min after illumination.

## References

[1] Genty B., Harbinson J., Briantais J.M., Baker N.R. (1990). The relationship between non-photochemical quenching of chlorophyll fluorescence and the rate of photosystem II photochemistry in leaves. Photosynth. Res..

[2] Ghashghaie J., Cornic G. (1994). Effect of temperature on partitioning of photosynthetic electron flow between CO_2_ assimilation and O_2_ reduction and on the CO_2_/O_2_ specificity of Rubisco. J. Plant Physiol..

[3] Ruuska S.A., Badger M.R., Andrews T.J., Von Caemmerer S. (2000). Photosynthetic electron sinks in transgenic tobacco with reduced amounts of Rubisco: Little evidence for significant Mehler reaction. J. Exp. Bot..

[4] Driever S.M., Baker N.R. (2011). The water–water cycle in leaves is not a major alternative electron sink for dissipation of excess excitation energy when CO_2_ assimilation is restricted. Plant Cell Environ..

[5] Miyake C. (2020). Molecular mechanism of oxidation of P700 and suppression of ROS production in photosystem I in response to electron-sink limitations in C3 plants. Antioxidants.

[6] Furutani R., Ifuku K., Suzuki Y., Noguchi K., Shimakawa G., Wada S., Makino A., Sohtome T., Miyake C., Hisabori T. (2020). P700 oxidation suppresses the production of reactive oxygen species in photosystem I. Advances in Botanical Research.

[7] Furutani R., Makino A., Suzuki Y., Wada S., Shimakawa G., Miyake C. (2020). Intrinsic fluctuations in transpiration induce photorespiration to oxidize P700 in photosystem I. Plants.

[8] Kadota K., Furutani R., Makino A., Suzuki Y., Wada S., Miyake C. (2019). Oxidation of P700 induces alternative electron flow in photosystem I in wheat leaves. Plants.

[9] Sejima T., Takagi D., Fukayama H., Makino A., Miyake C. (2014). Repetitive short-pulse light mainly inactivates photosystem I in sunflower leaves. Plant Cell Physiol..

[10] Hisabori T., Hisabori T. (2020). Regulation machineries of ATP synthase from phototroph. Advances in Botanical Research.

[11] Wada S., Suzuki Y., Miyake C. (2020). Photorespiration enhances acidification of the thylakoid lumen, reduces the plastoquinone pool, and contributes to the oxidation of P700 at a lower partial pressure of CO_2_ in wheat leaves. Plants.

[12] Avenson T.J., Cruz J.A., Kramer D.M. (2004). Modulation of energy-dependent quenching of excitons in antennae of higher plants. Proc. Natl. Acad. Sci. USA.

[13] Kanazawa A., Kramer D.M. (2002). In vivo modulation of nonphotochemical exciton quenching (NPQ) by regulation of the chloroplast ATP synthase. Proc. Natl. Acad. Sci. USA.

[14] Asada K. (2000). The water–water cycle as alternative photon and electron sinks. Philos. Trans. R. Soc. Lond. B Biol. Sci..

[15] Bang T.C., Husted S., Laursen K.H., Persson D.P., Schjoerring J.K. (2021). The molecular–physiological functions of mineral macronutrients and their consequences for deficiency symptoms in plants. New Phytol..

[16] Mattila H., Khorobrykh S., Havurinne V., Tyystjärvi E. (2015). Reactive oxygen species: Reactions and detection from photosynthetic tissues. J. Photochem. Photobiol. B Biol..

[17] Khorobrykh S., Havurinne V., Mattila H., Tyystjärvi E. (2020). Oxygen and ROS in photosynthesis. Plants.

[18] Sejima T., Hanawa H., Shimakawa G., Takagi D., Suzuki Y., Fukayama H., Makino A., Miyake C. (2016). Post-illumination transient O_2_-uptake is driven by photorespiration in tobacco leaves. Physiol. Plant..

[19] Zivcak M., Brestic M., Kunderlikova K., Sytar O., Allakhverdiev S.I. (2015). Repetitive light pulse-induced photoinhibition of photosystem I severely affects CO_2_ assimilation and photoprotection in wheat leaves. Photosynth. Res..

[20] Takahashi M.-A., Asada K. (1983). Superoxide anion permeability of phospholipid membranes and chloroplast thylakoids. Arch. Biochem. Biophys..

[21] Satoh K. (1970). Mechanism of photoinactivation in photosynthetic systems I. The dark reaction in photoinactivation1. Plant Cell Physiol..

[22] Satoh K. (1970). Mechanism of photoinactivation in photosynthetic systems II. The occurrence and properties of two different types of photoinactivation. Plant Cell Physiol..

[23] Satoh K. (1970). Mechanism of photoinactivation in photosynthetic systems III. Site and mode of photoinactivation in photosystem I. Plant Cell Physiol..

[24] Satoh K., Edelman M., Hallick R.B., Chua N.-H. (1982). Fractionation of thylakoid-bound chlorophyll-protein complexes by isoelectric focusing. Methods in Chloroplast Molecular Biology.

[25] Satoh K., Fork D.C. (1982). Photoinhibition of reaction centers of photosystems I and II in intact *Bryopsis* chloroplasts under anaerobic conditions. Plant Physiol..

[26] Inoue K., Sakurai H., Hiyama T. (1986). Photoinactivation sites of photosystem I in isolated chloroplasts. Plant Cell Physiol..

[27] Inoue K., Fujii T., Yokoyama E., Matsuura K., Hiyama T., Sakurai H. (1989). The photoinhibition site of photosystem I in isolated chloroplasts under extremely reducing conditions. Plant Cell Physiol..

[28] Havaux M., Davaud A. (1994). Photoinhibition of photosynthesis in chilled potato leaves is not correlated with a loss of Photosystem-II activity. Photosynth. Res..

[29] Sonoike K., Terashima I. (1994). Mechanism of photosystem-I photoinhibition in leaves of *Cucumis sativus* L.. Planta.

[30] Terashima I., Funayama S., Sonoike K. (1994). The site of photoinhibition in leaves of Cucumis sativus L. at low temperatures is photosystem I, not photosystem II. Planta.

[31] Ivanov A.G., Morgan R.M., Gray G.R., Velitchkova M.Y., Huner N.P.A. (1998). Temperature/light dependent development of selective resistance to photoinhibition of photosystem I. FEBS Lett..

[32] Tjus S.E., Lindberg Møller B., Vibe Scheller H. (1998). Photosystem I is an early target of photoinhibition in barley illuminated at chilling temperatures. Plant Physiol..

[33] Tjus S.E., Lindberg Møller B., Scheller H.V. (1999). Photoinhibition of photosystem I damages both reaction centre proteins PSI-A and PSI-B and acceptor-side located small photosystem I polypeptides. Photosynth. Res..

[34] Teicher H.B., Lindberg Møller B., Vibe Scheller H. (2000). Photoinhibition of photosystem I in field-grown barley (*Hordeum vulgare* L.): Induction, recovery and acclimation. Photosynth. Res..

[35] Tjus S.E., Scheller H.V., Andersson B., Møller B.L. (2001). Active oxygen produced during selective excitation of photosystem I is damaging not only to photosystem I, but also to photosystem II. Plant Physiol..

[36] Suorsa M., Järvi S., Grieco M., Nurmi M., Pietrzykowska M., Rantala M., Kangasjärvi S., Paakkarinen V., Tikkanen M., Jansson S. (2012). PROTON GRADIENT REGULATION5 is essential for proper acclimation of *Arabidopsis* photosystem I to naturally and artificially fluctuating light conditions. Plant Cell.

[37] Tikkanen M., Grieco M., Nurmi M., Rantala M., Suorsa M., Aro E.-M. (2012). Regulation of the photosynthetic apparatus under fluctuating growth light. Philos. Trans. R. Soc. B Biol. Sci..

[38] Suorsa M., Grieco M., Järvi S., Gollan P.J., Kangasjärvi S., Tikkanen M., Aro E.-M. (2013). PGR5 ensures photosynthetic control to safeguard photosystem I under fluctuating light conditions. Plant Signal. Behav..

[39] Suorsa M., Rossi F., Tadini L., Labs M., Colombo M., Jahns P., Kater M.M., Leister D., Finazzi G., Aro E.-M. (2016). PGR5-PGRL1-dependent cyclic electron transport modulates linear electron transport rate in *Arabidopsis thaliana*. Mol. Plant.

[40] Tiwari A., Mamedov F., Grieco M., Suorsa M., Jajoo A., Styring S., Tikkanen M., Aro E.M. (2016). Photodamage of iron-sulphur clusters in photosystem I induces non-photochemical energy dissipation. Nat. Plants.

[41] Miyake C., Asada K., Larkum A.W.D., Douglas S.E., Raven J.A. (2003). The water-water cycle in algae. Photosynthesis in Algae.

[42] Shigeoka S., Maruta T. (2014). Cellular redox regulation, signaling, and stress response in plants. Biosci. Biotech. Biochem..

[43] Maruta T., Sawa Y., Shigeoka S., Ishikawa T. (2016). Diversity and evolution of ascorbate peroxidase functions in chloroplasts: More than just a classical antioxidant enzyme?. Plant Cell Physiol..

[44] Noctor G., Reichheld J.-P., Foyer C.H. (2018). ROS-related redox regulation and signaling in plants. Semin. Cell Dev. Biol..

[45] Terai Y., Ueno H., Ogawa T., Sawa Y., Miyagi A., Kawai-Yamada M., Ishikawa T., Maruta T. (2020). Dehydroascorbate reductases and glutathione set a threshold for high-light–induced ascorbate accumulation. Plant Physiol..

[46] Shimakawa G., Miyake C. (2018). Oxidation of P700 ensures robust photosynthesis. Front. Plant Sci..

[47] Harbinson J., Hedley C.L. (1989). The kinetics of P700^+^ reduction in leaves: A novel in situ probe of thylakoid functioning. Plant Cell Environ..

[48] Harbinson J., Genty B., Baker N.R. (1990). The relationship between CO_2_ assimilation and electron transport in leaves. Photosynth. Res..

[49] Harbinson J., Foyer C.H. (1991). Relationships between the efficiencies of photosystems I and II and stromal redox state in CO_2_-free air. Plant Physiol..

[50] Harbinson J., Hedley C.L. (1993). Changes in P700 oxidation during the early stages of the induction of photosynthesis. Plant Physiol..

[51] Golding A.J., Johnson G.N. (2003). Down-regulation of linear and activation of cyclic electron transport during drought. Planta.

[52] Golding A.J., Joliot P., Johnson G.N. (2005). Equilibration between cytochrome f and P700 in intact leaves. Biochim. Biophys. Acta (BBA) Bioenergy.

[53] Miyake C., Horiguchi S., Makino A., Shinzaki Y., Yamamoto H., Tomizawa K.-I. (2005). Effects of light intensity on cyclic electron flow around PSI and its relationship to non-photochemical quenching of chl fluorescence in Tobacco Leaves. Plant Cell Physiol..

[54] Miyake C., Miyata M., Shinzaki Y., Tomizawa K.-I. (2005). CO_2_ response of cyclic electron flow around PSI (CEF-PSI) in tobacco leaves—Relative electron fluxes through PSI and PSII determine the magnitude of non-photochemical quenching (NPQ) of chl fluorescence. Plant Cell Physiol..

[55] Shimakawa G., Murakami A., Niwa K., Matsuda Y., Wada A., Miyake C. (2019). Comparative analysis of strategies to prepare electron sinks in aquatic photoautotrophs. Photosynth. Res..

[56] Van Maarschalkerweerd M., Husted S.R. (2015). Recent developments in fast spectroscopy for plant mineral analysis. Front. Plant Sci..

[57] Ma J.F., Tsay Y.-F. (2021). Transport systems of mineral elements in plants: Transporters, regulation and utilization. Plant Cell Physiol..

[58] Watanabe T., Azuma T. (2021). Ionomic variation in leaves of 819 plant species growing in the botanical garden of Hokkaido University, Japan. J. Plant Res..

[59] Klughammer C., Schreiber U. (2008). Saturation pulse method for assessment of energy conversion in PS I. PAM Appl. Notes.

[60] Baker N.R. (2008). Chlorophyll fluorescence: A probe of photosynthesis in vivo. Annu. Rev. Plant Biol..

[61] Miyake C., Amako K., Shiraishi N., Sugimoto T. (2009). Acclimation of tobacco leaves to high light intensity drives the plastoquinone oxidation system--relationship among the fraction of open PSII centers, non-photochemical quenching of chl fluorescence and the maximum quantum yield of PSII in the dark. Plant Cell Physiol..

[62] Makino A., Sakashita H., Hidema J., Mae T., Ojima K., Osmond B. (1992). Distinctive responses of ribulose-1,5-bisphosphate carboxylase and carbonic anhydrase in wheat leaves to nitrogen nutrition and their possible relationships to CO_2_-transfer resistance. Plant Physiol..

[63] Makino A., Osmond B. (1991). Effects of nitrogen nutrition on nitrogen partitioning between chloroplasts and mitochondria in pea and wheat. Plant Physiol..

[64] Ogawa Y., Katoh H., Ishikawa M. (1989). A Simple analytical method for index of soil nitrogen availability by extracting in phosphate buffer solution. (written in Japanese). J. Sci. Soil Manure.

[65] Truogh E. (1930). The determination of the readily available phosphorus of soils. J. Am. Soc. Agron..

[66] Helmke P.A., Sparks D.L., Bigham J.M. (1996). Lithium, sodium, potassium, rubidium, and cesium. Methods of Soil Analysis Part 3—Chemical Methods.

[67] Lindsay W.L., Norvell W.A. (1978). Development of a DTPA soil test for zinc, iron, manganese, and copper. Soil Sci. Soc. Am. J..

[68] Sherman G.D., Mchargue J.S., Hodgkiss W.S. (1942). Determination of active manganese in soil. Soil Sci..

[69] Tikhonov A.N. (2014). The cytochrome b_6_f complex at the crossroad of photosynthetic electron transport pathways. Plant Physiol. Biochem..

[70] Tikhonov A.N. (2018). The cytochrome b_6_f Complex: Biophysical aspects of its functioning in chloroplasts. Subcell. Biochem..

[71] Tikkanen M., Rantala S., Aro E.-M. (2015). Electron flow from PSII to PSI under high light is controlled by PGR5 but not by PSBS. Front. Plant Sci..

[72] Shaku K., Shimakawa G., Hashiguchi M., Miyake C. (2015). Reduction-induced suppression of electron flow (RISE) in the photosynthetic electron transport system of *Synechococcus elongatus* PCC 7942. Plant Cell Physiol..

[73] Shimakawa G., Shaku K., Miyake C. (2018). Reduction-induced suppression of electron flow (RISE) is relieved by non-ATP-consuming electron flow in *Synechococcus elongatus* PCC 7942. Front. Microbiol..

[74] Rantala S., Lempiäinen T., Gerotto C., Tiwari A., Aro E.M., Tikkanen M. (2020). PGR5 and NDH-1 systems do not function as protective electron acceptors but mitigate the consequences of PSI inhibition. Biochim. Biophys. Acta (BBA) Bioenergy.

[75] Malone L.A., Proctor M.S., Hitchcock A., Hunter C.N., Johnson M.P. (2021). Cytochrome b_6_f—Orchestrator of photosynthetic electron transfer. Biochim. Biophys. Acta (BBA) Bioenergy.

[76] Hernández I., Munné-Bosch S. (2015). Linking phosphorus availability with photo-oxidative stress in plants. J. Exp. Bot..

[77] Minagawa J. (2011). State transitions—The molecular remodeling of photosynthetic supercomplexes that controls energy flow in the chloroplast. Biochim. Biophys. Acta (BBA) Bioenergy.

[78] Barber J. (2008). Photosynthetic generation of oxygen. Philos. Tran. R. Soc. B Biol. Sci..

[79] Shitov A.V., Terentyev V.V., Zharmukhamedov S.K., Rodionova M.V., Karacan M., Karacan N., Klimov V.V., Allakhverdiev S.I. (2018). Is carbonic anhydrase activity of photosystem II required for its maximum electron transport rate?. Biochim. Biophys. Acta (BBA) Bioenergy.

[80] Lu Y.-K., Theg S.M., Stemler A.J. (2005). Carbonic anhydrase activity of the photosystem II OEC33 protein from pea. Plant Cell Physiol..

[81] Tsui E.Y., Agapie T. (2013). Reduction potentials of heterometallic manganese-oxido cubane complexes modulated by redox-inactive metals. Proc. Natl. Acad. Sci. USA.

[82] Saito K., Mandal M., Ishikita H. (2020). Energetics of ionized water molecules in the H-bond network near the Ca^2+^ and Cl^−^ binding sites in photosystem II. Biochemistry.

[83] Lohmiller T., Shelby M.L., Long X., Yachandra V.K., Yano J. (2015). Removal of Ca^2+^ from the oxygen-evolving complex in photosystem II has minimal effect on the Mn_4_O_5_ core structure: A polarized Mn X-ray absorption spectroscopy study. J. Physic. Chem. B.

[84] Michelet L., Zaffagnini M., Morisse S., Sparla F., Pérez-Pérez M.E., Francia F., Danon A., Marchand C.H., Fermani S., Trost P. (2013). Redox regulation of the Calvin–Benson cycle: Something old, something new. Front. Plant Sci..

[85] Tripp B.C., Smith K., Ferry J.G. (2001). Carbonic anhydrase: New insights for an ancient enzyme. J. Biol. Chem..

[86] Andrews T.J., Lorimer G.H., Hatch M.D., Boardman N.K. (1987). 3—Rubisco: Structure, mechanisms, and prospects for improvement. Photosynthesis.

[87] Tränkner M., Tavakol E., Jákli B. (2018). Functioning of potassium and magnesium in photosynthesis, photosynthate translocation and photoprotection. Physiol. Plant..

[88] Battie-Laclau P., Laclau J.-P., Beri C., Mietton L., Muniz M.R.A., Arenque B.C., De Cassia Piccolo M., Jordan-Meille L., Bouillet J.-P., Nouvellon Y. (2014). Photosynthetic and anatomical responses of *Eucalyptus grandis* leaves to potassium and sodium supply in a field experiment. Plant Cell Environ..

[89] Weng X.-Y., Zheng C.-J., Xu H.-X., Sun J.-Y. (2007). Characteristics of photosynthesis and functions of the water–water cycle in rice (*Oryza sativa*) leaves in response to potassium deficiency. Physiol. Plant..

[90] Jákli B., Tavakol E., Tränkner M., Senbayram M., Dittert K. (2017). Quantitative limitations to photosynthesis in K deficient sunflower and their implications on water-use efficiency. J. Plant Physiol..

[91] Droppa M., Horváth G. (1990). The role of copper in photosynthesis. CRC Crit. Rev. Plant Sci..

[92] Klughammer C., Schreiber U. (2016). Deconvolution of ferredoxin, plastocyanin, and P700 transmittance changes in intact leaves with a new type of kinetic LED array spectrophotometer. Photosynth. Res..

[93] Vassiliev I.R., Antonkine M.L., Golbeck J.H. (2001). Iron–sulfur clusters in type I reaction centers. Biochim. Biophys. Acta (BBA) Bioenergy.

[94] Baniulis D., Yamashita E., Zhang H., Hasan S.S., Cramer W.A. (2008). Structure-function of the cytochrome *b_6_f* complex. Photochem. Photobiol..

[95] Kroh G.E., Pilon M. (2020). Regulation of iron homeostasis and use in chloroplasts. Int. J. Mol. Sci..

[96] O'Neill M.A., Ishii T., Albersheim P., Darvill A.G. (2004). Rhamnogalacturonan II: Structure and function of a borate cross-linked cell wall pectic polysaccharide. Ann. Rev. Plant Biol..

[97] Kaiser B.N., Gridley K.L., Ngaire Brady J., Phillips T., Tyerman S.D. (2005). The role of molybdenum in agricultural plant production. Ann. Bot..

[98] Marschner H. (2012). Marschner’s Mineral Nutrition of Higher Plants.

