# Supplementary material for: Photosynthetic Parameters Show Specific Responses to Essential Mineral Deficiencies
Source: Antioxidants (Basel). 2021 Jun 23;10(7):996. doi: 10.3390/antiox10070996 (PMC8300717; doi:10.3390/antiox10070996)
Supplement: Supplementary file 1 [file antioxidants-10-00996-s001.zip › antioxidants-1234631-supplementary.pdf]

Supplementary Figure S1

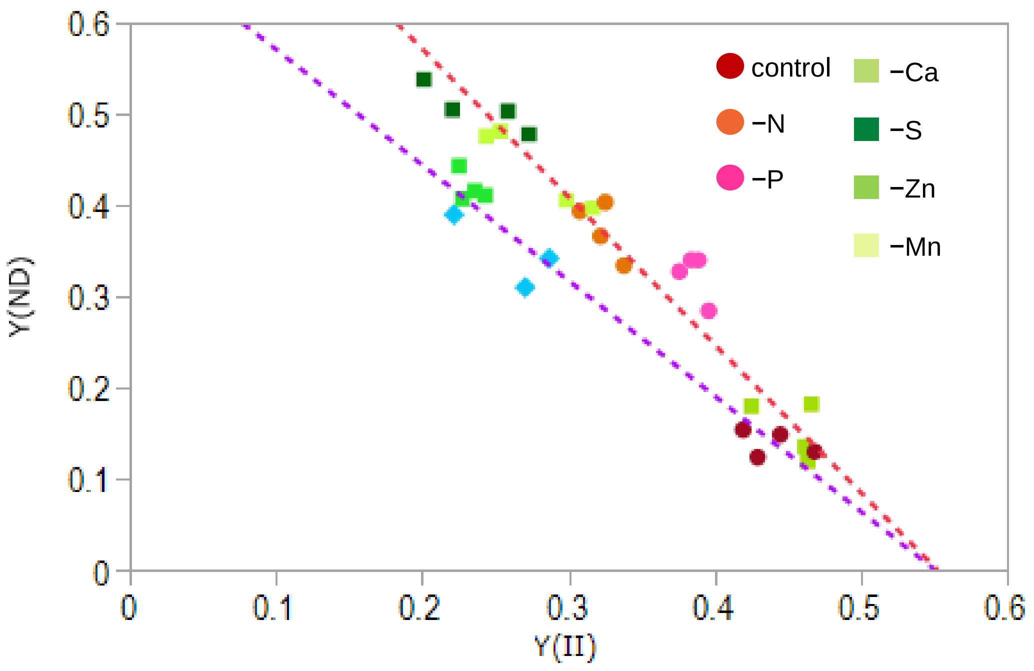

grp			LSM
High	a		0.3183
Middle		b	0.2530

grp	-grp	ΔLSM	SE	t value	p value (Prob>|t|)	CI (95 %)	
Middle	High	-0.07	0.01	-4.81	<.0001	-0.09	-0.04

Grp; group, CI; Confidential Interval, LSM; the least square means
ΔLSM; the difference of the least square means, SE; Standard error

Regression line of "High" group, consists of "control", "−N", "−P", "−Mn" and "− S"
y = −1.622x + 0.896 (R^2 = 0.931)

Regression line of "Middle" group, consists of "control", "−Zn" and "−Mg"
y = − 1.267x + 0.698 (R^2 = 0.963)

Supplementary Figure S1 Comparison of the dependence of Y(ND) on Y(II) in the intact leaves of sunflower plants grown hidroponically. The dependency of Y(ND) on Y(II) (Figure 2) was evaluated by comparing the slopes of the regression lines in the covariance analysis (ANCOVA). The data distributed in both the high P700 oxidation and middle oxidation areas was presented and analyzed in Figure 2. We arbitrarily divided the nutrient-deficiency plants into "High" and "Middle" groups, which included the control conditions "Control", nitrogen deficiency "−N", phosphate deficiency "−P", manganese deficiency "−Mn", sulfur deficiency "−S" in the "High" group, and the "Control", zinc deficiency "−Zn", magnesium deficiency "−Mg " . The control group was included in both the "High" and "Middle" group, as the "Control" condition was located with the common origin of higher Y(II). The magnitudes of the differences (ΔLSM, difference of least square mean) between the slopes of the "High" and "Middle" groups were compared. The "High" group statistically showed a higher value when compared to that in the "Middle" group.

Supplementary Figure S2

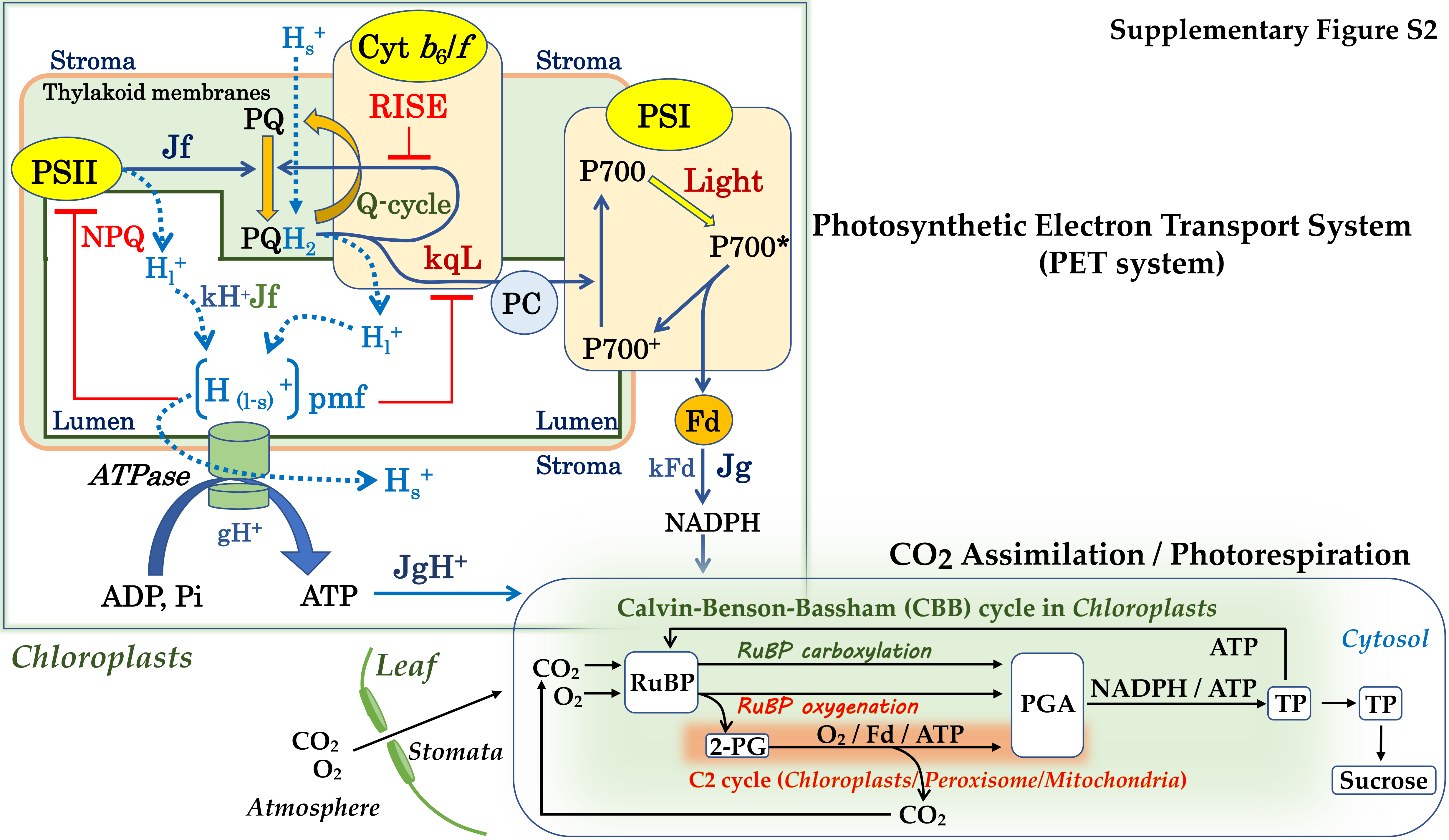

Supplementary Figure S2 Regulation of the electron and H^+ fluxes in photosynthesis in response to the activities of both CO_2 assimilation and photorespiration in C3 plants. In C3-photosynthesis, both CO_2 assimilation and photorespiration proceed. CO_2 assimilation is driven by both the photosynthetic electron transport (PET) system and the Calvin-Benson-Bassham (CBB) cycle in chloroplasts. Photorespiration is driven cooperatively by the PET system, the CBB-cycle, and the C2-cycle to regenerate ribulose 1,5-bisphospate (RuBP), which is a substrate of RuBP carboxylase/oxygenase (Rubisco). The C2-cycle functions over three organelles, including chloroplasts, peroxisomes, and mitochondria.

In the PET system, both photosystem (PS) II (PSII) and PSI absorb light energy to excite both the reaction center chlorophylls, P680 in PSII and P700 in PSI. Both P680 and P700 turnover in the photo-oxidation reduction cycles are as follows: P680 => P680* => $P680^+$ => P680 in PSII; P700 => P700* => $P700^+$ => P700 in PSI. Both P680* and P700* were produced in the photo-excitations of P680 and P700. Both $P680^+$ and $P700^+$ are produced by the oxidation of P680* and P700* by their electron acceptors, plastoquinone (PQ) in PSII and ferredoxin (Fd) in PSI. Finally, the ground-states of P680 and P700 are regenerated by the reduction of $P680^+$ and $P700^+$ by the electrons from H_2O in PSII and plastocyanin (PC) in PSI. The oxidized PQ is regenerated during the oxidation of the reduced PQ (PQH_2) by the cytochrome (Cyt) b_6/f-complex, and the reduced PC is regenerated by the reduction of the PC by the Cyt b_6/f-complex. The oxidized Fd is regenerated in the oxidation reaction of the reduced Fd catalyzed by Fd-NADP oxidoreductase to produce NADPH. The summation of the above sequential reaction from PSII to NADPH constitutes the electron transport reaction in the PET system, which is called photosynthetic linear electron flow. Simultaneously with photosynthetic linear electron flow, H^+ accumulates in the lumen of the thylakoid membranes by the oxidation of H_2O to (O_2 + e^- + H^+) in PSII and Q-cycle turnover of PQ in the Cyt b_6/f-complex. The accumulation of H^+ produces a proton motive force (pmf) that drives the ATP synthase of the thylakoid membranes to produce ATP. In other words, photosynthetic linear electron flow produces both NADPH and ATP in the PET system.

CO_2 assimilation uses both NADPH and ATP as chemical energy compounds to fix CO_2, which enters from the atmosphere into chloroplasts through the stomata, and regenerates RuBP in the CBB cycle in chloroplasts. Rubisco catalyzes the carboxylation of RuBP using CO_2 to produce 3-phosphoglyceric acid (PGA). A portion of the carbon in PGA is exported to the cytosol from the chloroplast to be converted to sucrose. Then, the remaining carbon originating from PGA is used for the regeneration of RuBP using both NADPH and ATP. The CB cycle can then continue to assimilate CO_2.

Photorespiration starts with the oxygenation of RuBP by RuBisCO using O_2 with the production of both PGA and 2-phosphoglycolate (2-PG) under atmospheric conditions. PGA enters the CB cycle and is converted to RuBP. 2-PG is converted to PGA in the C2-cycle; however, 75% of the carbon of 2-PG is recovered as PGA, which is further used for the regeneration of RuBP in the CBB cycle. The remaining carbon (25%) from the PGA is released as CO_2, which is fixed in the CBB cycle to regenerate RuBP. Both the C2-cycle and the CBB-cycle use NADPH, ATP, and Fd to regenerate RuBP for photorespiration.

In C3-photosynthesis under atmospheric conditions, the PET system functions in a mode tightly coupled with CO_2 assimilation and photorespiration (Furutani et al. 2020a, b, Miyake 2020). Almost all electrons and ATP, both of which are produced in photosynthetic linear electron flow, are used by both CO_2 assimilation and photorespiration. That is, photosynthetic linear electron flow is driven by both CO_2 assimilation and photorespiration, and the reverse is also true. The tight coupling of the PET system with both CO_2 assimilation and photorespiration is supported by the fact that the redox reaction of Fd is mainly driven by the photosynthetic linear electron flow, the CB cycle, and the C2-cycle (Kadota et al. 2019).

Supplementary Table S1

	P g/kg	K g/kg	Ca g/kg	Mg g/kg	S g/kg	Mn mg/kg	Cu mg/kg	Fe mg/kg	Zn mg/kg	Mo mg/kg	B mg/kg
control	10.3 ± 0.2	37 ± 5	16.8 ± 0.4	4.3 ± 0.4	9.0 ± 0.5	284 ± 15	9.9 ± 0.5	60 ± 10	33 ± 7	5.4 ±0.3	62 ±4
N free	4.50 ± 0.3	39 ± 3	14.6 ± 0.3	2.91 ± 0.09	4.7 ± 1.1	250 ± 16	7.6 ± 0.8	32 ± 5	21 ± 8	3.02 ±0.13	79 ±8
P free	2.30 ± 0.3	34 ± 2	13.2 ± 1.2	3.3 ± 0.3	6.64 ± 0.14	220 ± 40	14.7 ± 0.7	73 ± 4	60 ± 7	5.1 ±0.6	37 ±3
K free	17 ± 3	7 ± 1	20.1 ± 0.2	10.0 ± 1.5	7.67 ± 0.16	490 ± 30	7.7 ± 1.0	88 ± 13	37 ± 6	7.1 ±0.8	74 ±6
Ca free	9.3 ± 0.8	36.7 ± 1.4	1.1 ± 0.2	10.7 ± 0.1	6.0 ± 0.5	740 ± 50	8.6 ± 1.7	35 ± 6	51 ± 14	4.4 ±0.3	59 ±13
Mg free	7.9 ± 0.6	48 ± 5	19.9 ± 1.3	0.52 ± 0.05	5.4 ± 1.2	522 ± 14	11.2 ± 0.7	48 ± 5	41 ± 3	5.0 ±0.3	68 ±4
S free	9.5 ± 0.3	31.2 ± 1.7	12.2 ± 0.8	4.2 ± 0.2	3.7 ± 0.9	200 ± 30	10.0 ± 1.0	47 ± 11	30 ± 10	100 ±8	75 ±3
Mn free	12.5 ± 0.5	40.6 ± 1.1	14.5 ± 0.8	4.70 ± 0.05	7.4 ± 0.5	12 ± 3	7.25 ± 0.11	78 ± 8	30 ± 6	5.0 ±0.5	62 ±6
Cu free	9.0 ± 0.6	40 ± 3	12.9 ± 0.8	4.1 ± 0.2	8.5 ± 1.3	310 ± 40	0.56 ± 0.16	82 ± 5	35 ± 2	6.0 ±0.3	71 ±3
Fe free	9.5 ± 0.3	46.0 ± 1.8	15.0 ± 0.4	5.30 ± 0.15	7.31 ± 0.10	460 ± 50	18.0 ± 1.8	30 ± 5	111 ± 13	5.5 ±0.7	69 ±2
Zn free	34.2 ± 0.4	36 ± 3	23 ± 2	7.9 ± 0.6	7.7 ± 0.6	420 ± 20	5.7 ± 0.5	76 ± 9	4 ± 2	4.26 ±0.19	75 ±5
Mo free	12.4 ± 0.6	33.6 ± 0.3	18.0 ± 1.1	5.9 ± 0.8	7.9 ± 0.6	400 ± 40	7.8 ± 1.6	91 ± 11	34 ± 9	0.30 ±0.05	76 ±4
B free	11.1 ± 0.7	42.6 ± 1.2	14.3 ± 1.4	5.02 ± 0.17	8.5 ± 0.3	280 ± 30	6.8 ± 0.8	80 ± 30	41 ± 2	4.6 ±0.4	42 ±3

Elemental analysis data are shown as mean ± SD. The number of biological replicates was 3.

Supplementary Table S2

control

decreased ▽ increased

Trt	Chlorophyll (mmol/m^2)				Nitrogen (mmol/m^2)				Fv/Fm				Pm (Relative value)			
	grp	mean	SD	CI	grp	mean	SD	CI	grp	mean	SD	CI	grp	mean	SD	CI
control	I	0.4	0.04	(0.36, 0.44)	I	109	10	(98, 120)	I	0.833	0.003	(0.829, 0.836)	I	1.27	0.11	(1.15, 1.40)
Ca free		0.28	0.07	(0.20, 0.36)		135	4	(130, 140)	I	0.823	0.004	(0.819, 0.828)	I	0.9	0.3	(0.6, 1.3)
B free		0.289	0.005	(0.284, 0.295)	I	97	9	(87, 108)	I	0.835	0.005	(0.829, 0.840)	I	1.1	0.03	(1.07, 1.14)
Cu free	I	0.37	0.05	(0.32, 0.43)	I	127	12	(113, 140)	I	0.789	0.01	(0.778, 0.801)	I	1.4	0.03	(1.36, 1.44)
Fe free		0.13	0.03	(0.10, 0.17)	I	100	14	(84, 117)	I	0.812	< 0.01	(0.811, 0.812)		0.26	0.1	(0.14, 0.37)
K free	I	0.362	0.018	(0.341, 0.382)		132	2	(130, 134)	I	0.825	0.003	(0.821, 0.828)	I	1.23	0.04	(1.19, 1.28)
Mg free		0.3	0.03	(0.26, 0.33)	I	112	7	(104, 119)		0.77	0.04	(0.73, 0.82)		0.9	0.2	(0.7, 1.1)
Mn free		0.27	0.05	(0.21, 0.32)	I	105	8	(96, 115)		0.68	0.06	(0.60, 0.75)		0.9	0.04	(0.86, 0.94)
Mo free	I	0.39	0.02	(0.366, 0.414)	I	119	7	(111, 127)	I	0.835	0.006	(0.829, 0.842)	I	1.3	0.2	(1.0, 1.5)
N free		0.245	0.003	(0.242, 0.248)		67	6	(61, 74)	I	0.818	0.008	(0.809, 0.826)		0.76	0.08	(0.67, 0.85)
P free	I	0.46	0.05	(0.40, 0.51)	I	125	5	(119, 130)	I	0.815	0.005	(0.809, 0.820)	I	1.22	0.14	(1.06, 1.40)
S free		0.24	0.03	(0.21, 0.26)	I	123	9	(113, 134)	I	0.816	0.017	(0.797, 0.836)		0.74	0.08	(0.58, 090)
Zn free	I	0.39	0.04	(0.34, 0.44)	I	93	7	(85, 101)	I	0.822	0.006	(0.815, 0.828)	I	1.5	0.3	(1.0, 2.1)

Trt, Mineral deficiency treatment; CI, Confidential Interval (95%); SD, Standard Deviation; The number of biological replicates = 3; grp, Comparative grouping by Dunnett's-test ($p<0.05$). No symbol represents the significant difference against control treatment. Group I shows significantly the same with the control treatment.

Supplementary Table S3

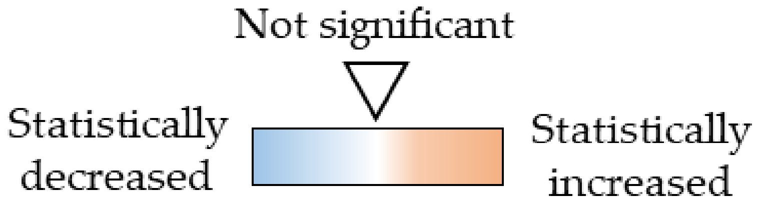

Type I

Time (min)	-N Y(I)	-N Y(ND)	-N Y(NA)	-N Y(II)	-P Y(I)	-P Y(ND)	-P Y(NA)	-P Y(II)
0	0.072	-	0.072	0.001**	<0.001**	-	<0.001**	0.194
2.5	0.568	0.485	0.891	0.017*	0.232	0.282	0.250	0.957
5	<0.001**	<0.001**	0.418	0.003**	0.166	<0.001**	<0.001**	0.018*
7.5	<0.001**	<0.001**	0.966	<0.001**	0.003**	<0.001**	<0.001**	<0.001**
10	<0.001**	<0.001**	0.332	<0.001**	0.009**	<0.001**	<0.001**	0.003**

Type II

Time (min)	-Ca Y(I)	-Ca Y(ND)	-Ca Y(NA)	-Ca Y(II)	-S Y(I)	-S Y(ND)	-S Y(NA)	-S Y(II)	-Zn Y(I)	-Zn Y(ND)	-Zn Y(NA)	-Zn Y(II)	-Mn Y(I)	-Mn Y(ND)	-Mn Y(NA)	-Mn Y(II)
0	0.112	-	0.112	0.048*	0.045*	-	0.045*	<0.001**	0.005**	-	0.005**	0.052	<0.001**	-	<0.001**	0.241
2.5	<0.001**	<0.001**	<0.001**	0.003**	0.003**	<0.001**	<0.001**	0.262	0.002**	0.003 **	0.002**	0.012*	0.003**	<0.001**	<0.001**	0.729
5	0.086	0.141	0.413	0.012*	<0.001**	<0.001**	0.008**	<0.001**	<0.001**	<0.001**	0.016*	<0.001**	0.002**	<0.001**	0.304	0.009**
7.5	0.002**	0.707	0.064	0.307	<0.001**	<0.001**	0.001**	<0.001**	<0.001**	<0.001**	0.162	<0.001**	<0.001**	<0.001**	0.274	<0.001**
10	0.001**	0.272	0.104	0.560	<0.001**	<0.001**	<0.001**	<0.001**	<0.001**	<0.001**	0.789	<0.001**	<0.001**	<0.001**	0.080	<0.001**

Type III

Time (min)	-Mo Y(I)	-Mo Y(ND)	-Mo Y(NA)	-Mo Y(II)	-B Y(I)	-B Y(ND)	-B Y(NA)	-B Y(II)	-Cu Y(I)	-Cu Y(ND)	-Cu Y(NA)	-Cu Y(II)
0	0.212	-	0.212	0.391	0.648	-	0.648	0.597	<0.001**	-	<0.001**	0.002**
2.5	0.010**	0.356	0.054	<0.001**	0.015*	0.356	0.071	0.001**	<0.001**	0.615	0.007**	<0.001**
5	<0.001**	<0.001**	<0.001**	<0.001**	<0.001**	<0.001**	<0.001**	<0.001**	<0.001**	<0.001**	<0.001**	<0.001**
7.5	0.001**	0.080	0.004**	<0.001**	<0.001**	<0.001**	<0.001**	<0.001**	<0.001**	0.383	<0.001**	<0.001**
10	0.002**	0.010*	0.001**	0.001**	<0.001**	0.098	<0.001**	<0.001**	<0.001**	0.005**	<0.001**	<0.001**

Time (min)	-K Y(I)	-K Y(ND)	-K Y(NA)	-K Y(II)	-Mg Y(I)	-Mg Y(ND)	-Mg Y(NA)	-Mg Y(II)	-Fe Y(I)	-Fe Y(ND)	-Fe Y(NA)	-Fe Y(II)
0	0.003**	-	0.003**	0.118	0.140	-	0.140	<0.001**	0.007**	-	0.007**	<0.001**
2.5	0.260	0.485	0.367	0.349	0.923	0.210	0.459	0.001**	0.249	0.356	0.851	<0.001**
5	0.013*	0.005**	0.006**	0.060	<0.001**	0.205	0.002**	<0.001**	<0.001**	<0.001**	<0.001**	<0.001**
7.5	0.031*	0.099	0.027*	0.128	<0.001**	<0.001**	0.033*	<0.001**	<0.001**	0.011*	<0.001**	<0.001**
10	0.018*	0.128	0.022*	0.107	<0.001**	<0.001**	0.051	<0.001**	<0.001**	0.007**	<0.001**	<0.001**

The statistical difference in each original parameter between control and nutrients-deficient treated sunflower leaves were estimated by Student's t-test. *p* values are shown in the table. A *p* value less than 0.05 was assumed to be statistically significant. *; $p < 0.05$, **; $p < 0.01$. Hyphen (-) shows that the values of the parameters taken from both control and nutrients-deficient treated plants were zero. Data are the same as those used in Figure 3 and 5. The number of biological replicate was 4.

Supplementary Table S4

Not significant

Statistically decreased — Statistically increased

Type I

Time (min)	-N				-P			
	Y(ND)/Y(II)$_N$	Y(NA)/Y(II)$_N$	Y(NPQ)/Y(II)$_N$	(1-qL)/Y(II)$_N$	Y(ND)/Y(II)$_N$	Y(NA)/Y(II)$_N$	Y(NPQ)/Y(II)$_N$	(1-qL)/Y(II)$_N$
0	-	0.356	<0.001**	0.356	-	0.045*	<0.001**	0.049*
2.5	0.906	0.137	0.457	0.081	0.275	0.702	0.363	0.951
5	0.383	0.116	0.241	0.069	0.045*	0.010*	0.089	0.294
7.5	0.008**	0.129	0.011*	0.250	<0.001**	<0.001**	0.027*	0.054
10	0.002**	0.076	0.011*	0.351	<0.001**	0.002**	0.165	0.267

Type II

Time (min)	-Ca				-S			
	Y(ND)/Y(II)$_N$	Y(NA)/Y(II)$_N$	Y(NPQ)/Y(II)$_N$	(1-qL)/Y(II)$_N$	Y(ND)/Y(II)$_N$	Y(NA)/Y(II)$_N$	Y(NPQ)/Y(II)$_N$	(1-qL)/Y(II)$_N$
0	-	0.356	<0.001**	0.356	-	0.356	0.541	0.356
2.5	<0.001**	<0.001**	<0.001**	<0.001**	<0.001**	<0.001**	0.823	<0.001**
5	0.001**	0.129	0.211	0.004**	0.050*	0.004**	<0.001**	0.002**
7.5	0.014*	0.617	0.918	0.016*	0.047*	<0.001**	<0.001**	0.004**
10	0.062	0.674	0.910	0.041*	0.005**	<0.001**	<0.001**	0.002**

Time (min)	-Zn				-Mn			
	Y(ND)/Y(II)$_N$	Y(NA)/Y(II)$_N$	Y(NPQ)/Y(II)$_N$	(1-qL)/Y(II)$_N$	Y(ND)/Y(II)$_N$	Y(NA)/Y(II)$_N$	Y(NPQ)/Y(II)$_N$	(1-qL)/Y(II)$_N$
0	-	0.201	<0.001**	0.123	-	0.454	<0.001**	0.457
2.5	0.002**	0.045*	0.098	0.237	<0.001**	0.005**	0.831	0.993
5	0.016*	0.001**	<0.001**	<0.001**	0.005**	0.373	0.116	0.304
7.5	<0.001**	<0.001**	<0.001**	<0.001**	0.971	0.039*	0.016*	0.031*
10	<0.001**	0.001**	<0.001**	<0.001**	0.063	0.051	0.014*	0.021*

Type III

Time (min)	-Mo				-B			
	Y(ND)/Y(II)$_N$	Y(NA)/Y(II)$_N$	Y(NPQ)/Y(II)$_N$	(1-qL)/Y(II)$_N$	Y(ND)/Y(II)$_N$	Y(NA)/Y(II)$_N$	Y(NPQ)/Y(II)$_N$	(1-qL)/Y(II)$_N$
0	-	0.058	<0.001**	0.056	-	0.156	<0.001**	0.170
2.5	0.356	0.006**	0.128	0.006**	0.356	0.010*	0.023*	0.011*
5	<0.001**	<0.001**	<0.001**	<0.001**	<0.001**	<0.001**	<0.001**	<0.001**
7.5	<0.001**	0.031*	0.012*	0.026*	0.800	0.001**	0.001**	<0.001**
10	0.013*	0.014*	0.448	0.060	<0.001**	<0.001**	0.427	<0.001**

Time (min)	-Cu				-K			
	Y(ND)/Y(II)$_N$	Y(NA)/Y(II)$_N$	Y(NPQ)/Y(II)$_N$	(1-qL)/Y(II)$_N$	Y(ND)/Y(II)$_N$	Y(NA)/Y(II)$_N$	Y(NPQ)/Y(II)$_N$	(1-qL)/Y(II)$_N$
0	-	0.153	<0.001**	0.169	-	0.465	<0.001**	0.635
2.5	0.038*	0.003**	0.005**	0.002**	0.145	0.029*	0.245	0.024*
5	0.035*	<0.001**	<0.001**	<0.001**	0.080	0.006**	0.002**	0.970
7.5	<0.001**	<0.001**	<0.001**	<0.001**	0.022*	0.026*	0.006**	0.709
10	<0.001**	<0.001**	0.004**	<0.001**	0.013*	0.017*	0.004**	0.730

Time (min)	-Mg				-Fe			
	Y(ND)/Y(II)$_N$	Y(NA)/Y(II)$_N$	Y(NPQ)/Y(II)$_N$	(1-qL)/Y(II)$_N$	Y(ND)/Y(II)$_N$	Y(NA)/Y(II)$_N$	Y(NPQ)/Y(II)$_N$	(1-qL)/Y(II)$_N$
0	-	0.356	0.020*	0.356	-	0.415	0.097	0.356
2.5	0.185	0.058	0.660	0.040*	0.356	0.002**	0.021*	<0.001**
5	0.912	0.013*	<0.001**	0.023*	0.002**	0.515	0.622	<0.001**
7.5	0.004**	0.105	0.004**	0.124	0.020*	0.019*	0.747	<0.001**
10	0.017*	0.989	0.194	0.330	0.406	0.003**	0.752	<0.001**

The statistical difference in each normalized parameter between control and nutrients-deficient treated sunflower leaves were estimated by Student's t-test. *p* values are shown in the table. A p value less than 0.05 was assumed to be statistically significant. *; $p < 0.05$, **; $p < 0.01$. Hyphen (-) shows that the values of the parameters taken from both control and nutrients-deficient treated plants were zero. Data are the same as those used in Figure 4 and 6. The number of biological replicate was 4.
